# Severe outcomes of individual or multiple respiratory viral infections in a large national healthcare system, 2022–2023

**DOI:** 10.3389/fpubh.2026.1808528

**Published:** 2026-04-22

**Authors:** Janet M. Grubber, Ikwo K. Oboho, Kaitlin N. Swinnerton, Theodore C. Feldman, Nhan V. Do, Nathanael R. Fillmore, Westyn Branch-Elliman, Paul A. Monach

**Affiliations:** 1 VA Cooperative Studies Program, Boston, MA, United States; 2 VA Boston Healthcare System, Boston, MA, United States; 3 Division of Infectious Diseases and Geographic Medicine, University of Texas Southwestern Medical Center, Dallas, TX, United States; 4 Veterans Affairs North Texas Health Care System, Dallas, TX, United States; 5 Harvard Medical School, Boston, MA, United States; 6 School of Medicine, Boston University, Boston, MA, United States; 7 Department of Medicine, Section of Infectious Diseases, Greater Los Angeles VA Healthcare System, Centre for Healthcare Innovation, Implementation, and Policy (CSHIIP), Los Angeles, CA, United States; 8 Los Angeles David Geffen School of Medicine, University of California, Los Angeles, Los Angeles, CA, United States

**Keywords:** COVID-19, hypoxemia, influenza, mortality, outcomes, respiratory syncytial virus, SARS-CoV-2

## Abstract

**Background:**

Routine testing for SARS-CoV-2, influenza, and respiratory syncytial virus (RSV) was deployed in a large US healthcare system in 2022–2023. This policy allowed identification of a large cohort of co-infected patients and comparison of outcomes without confounding by testing indication.

**Methods:**

Patients “triple-tested” in the US Veterans Health Administration were classified by infection status in the first week of a positive test. Multivariable logistic regression was used to estimate associations of different infections with hypoxemia (SpO_2_ < 90% or supplementary oxygen >2 L/min) or death, separately, expressed as adjusted odds ratios (aOR) with 95% confidence intervals (CI).

**Findings:**

Among 835,987 triple-tested patients, 170,592 (20.4%) tested positive for SARS-CoV-2 alone, 30,454 (3.6%) influenza alone, 13,207 (1.6%) RSV alone, and 1,300 (0.2%) multiple viruses. Frequencies of hypoxemia and death were 8.0 and 1.9% with SARS-CoV-2, 7.7 and 0.8% with influenza, 9.3 and 1.1% with RSV, 8.7 and 1.5% with multiple viruses, and 8.9 and 2.0% with all-negative tests. After adjustment for age and immune-suppressive drugs, odds of hypoxemia were slightly higher with influenza (aOR = 1.12, CI 1.06–1.17), lower with RSV (aOR = 0.91, CI 0.85–0.97), and not significantly different with multiple viruses (aOR = 1.09, CI 0.89–1.34), relative to SARS-CoV-2 alone. Odds of death were lower with influenza (aOR = 0.52, CI 0.46–0.60) or RSV (aOR = 0.51, CI 0.43–0.60) and no different with multiple infections (aOR = 0.86, CI 0.54–1.36), relative to SARS-CoV-2 alone.

**Interpretation:**

Co-infection was rare (0.2% of tested cases), with incidences of hypoxemia and death similar to SARS-CoV-2 alone. Death was less frequent with influenza or RSV than SARS-CoV-2.

## Introduction

Clinical severity of SARS-CoV-2 infection dropped dramatically with an increase in population-level immunity and predominance of variants derived from the omicron strain (1); however, adverse outcomes appear to remain more common following infection with SARS-CoV-2 than with either influenza or respiratory syncytial virus (RSV) (2–4). Symptom-based screening for all three viruses was available throughout the pandemic, however, screening testing policies (e.g., testing of asymptomatic patients admitted for other reasons) was much more common for SARS-CoV-2 than for other respiratory viruses. For this reason, many of these studies, despite adjustment for many variables, may be susceptible to residual confounding by indication.

Due in part to concerns about possible strain on hospital systems in the likely event of simultaneous increases in SARS-CoV-2, influenza, and RSV infections, sometimes termed a “triple-demic,” some hospital systems expanded inpatient screening policies to include testing patients for all three viruses simultaneously regardless of the presence of symptoms. These policies provide an opportunity to compare severity of outcomes in a uniformly tested population and also to evaluate the frequency and severity of co-infection with multiple viruses. Co-infection with SARS-CoV-2 and other respiratory viruses, especially influenza, has been recognized since the beginning of the pandemic and was associated with a higher risk of severe respiratory disease (but not mortality) in data from 2020 (5, 6). There have been few studies examining clinical outcomes associated with multiple infections since the development of SARS-CoV-2 vaccines (7) or the evolution of the Omicron variants (8).

In the primary analysis in the current study, we used a cohort of “triple-tested” patients to estimate and compare incidences of adverse outcomes (hypoxemia and mortality, separately). We also analyzed data from patients who tested positive for more than one virus in the same week, to assess the impact of co-infection on these outcomes.

## Materials and methods

### Data sources, participants, and study design

Data were obtained from the VA COVID-19 Shared Data Resource (9) and the Corporate Data Warehouse (CDW), which collates electronic health record (EHR) data from VA facilities nationwide. All Veteran patients who were tested during the same week for SARS-CoV-2, influenza A or A/B, and RSV (PCR or antigen), between January 1, 2022, and December 31, 2023, were included. Only results from the first triple-tested week with positive result(s) were used for analyses—regardless of whether future triple-tested positive results existed for the patient during the study period. Patients were classified based on the results of the first positive test as: SARS-CoV-2 only, influenza only, RSV only, SARS-CoV-2 and influenza, SARS-CoV-2 and RSV, influenza and RSV, or positive for all three. Among patients with no positive tests, the first triple-tested all-negative result week was used in analyses, so data were skewed to earlier time-points. Each triple-tested patient was included only once, linked to one triple-tested week. The earliest date from that week (if tests were done on multiple days during the same week) was deemed the index date.

### Primary outcomes

Outcomes included hypoxemia within 14 days or death within 4 weeks of the index date. Hypoxemia was defined as documented SpO_2_ < 90% or receipt of supplemental oxygen > 2 L/min. Cause of death data were not available.

### Patient clinical and demographic characteristics

Candidate predictors of severe disease were based on our previous work in the VA patient population (10). These included age, sex, SARS-CoV-2 vaccination and vaccine manufacturer, prior SARS-CoV-2 infection, body mass index, treatment with immune-suppressive drugs before infection, and comorbidities defined as per the Chronic Conditions Warehouse (CCW) (11) with some additional grouping, e.g., of cardiac diseases or cancers. Comorbidities were ascertained during the 1-year period before the index date. Immune-suppressive drug usage (which did not include chemotherapy) was defined based on documented intravenous administrations or dispensing of outpatient prescriptions during pre-specified time periods before the index date (1–18 months depending on the expected duration of immune-suppression from the drug). See Supplementary Table 1 for data definitions.

### Statistical analyses

The primary analyses were limited to patients who tested positive for viral infection to allow comparison of the odds and risk differences of severe adverse outcomes by viral infection type. Multivariable logistic regression was used to model the association between the clinical outcomes (hypoxemia or death, analyzed separately) as dependent variables and type of viral infection and covariates as independent variables. Viral infection was modeled as one 4-level variable: influenza, RSV, or multiple infections vs. SARS-CoV-2 (reference). Results were expressed as adjusted odds ratios (aOR) and adjusted risk differences (aRD), both with 95% confidence intervals (CI).

Covariates were chosen for multiple logistic regression in two ways in parallel. In one approach, clinical and demographic variables, chosen on the basis of association with severe COVID-19 in previous work (10), were assessed for association with the outcome of hypoxemia or death and, if significantly associated individually, were then included in a multivariable model. The second approach focused on identifying confounding variables for the association of infection type (SARS-CoV-2, influenza, RSV, or multiple) with the outcomes of hypoxemia or death. Numerous clinical and demographic variables were added to these model individually, to determine whether they changed the OR for the association of any viral infection response level with the adverse outcome by >10% (12). Variables that met this criterion were included in a final multivariable model along with the viral infection variable.

Once confounding variables were identified, models containing interaction terms between these variables and the infection variable were used to identify significant interactions. Stratified logistic regression models were then used to further assess potential effect modification based on significant interactions.

In secondary analyses, multivariable logistic regression was used to calculate aOR for the association between selected clinical and demographic variables (independent) and hypoxemia and death (dependent), separately, stratified by infection status (SARS-CoV-2 alone, influenza alone, RSV alone, multiple infections, or none). Age was included in the models as a 3-part spline.

Analyses were performed using SAS© software (version 9.4 M6, Statistical Package 15.3), accessed via SAS Enterprise Guide (version 8.3).

### Ethical considerations

Prior to data collection and analysis, this study was approved by the VA Boston Research and Development Committee as exempt human subjects research with a waiver of informed consent.

### Role of the funding source

The funding sources had no involvement in study design; collection, analysis, or interpretation; writing of the report; nor the decision to submit the paper for publication.

## Results

**Figure 1:**
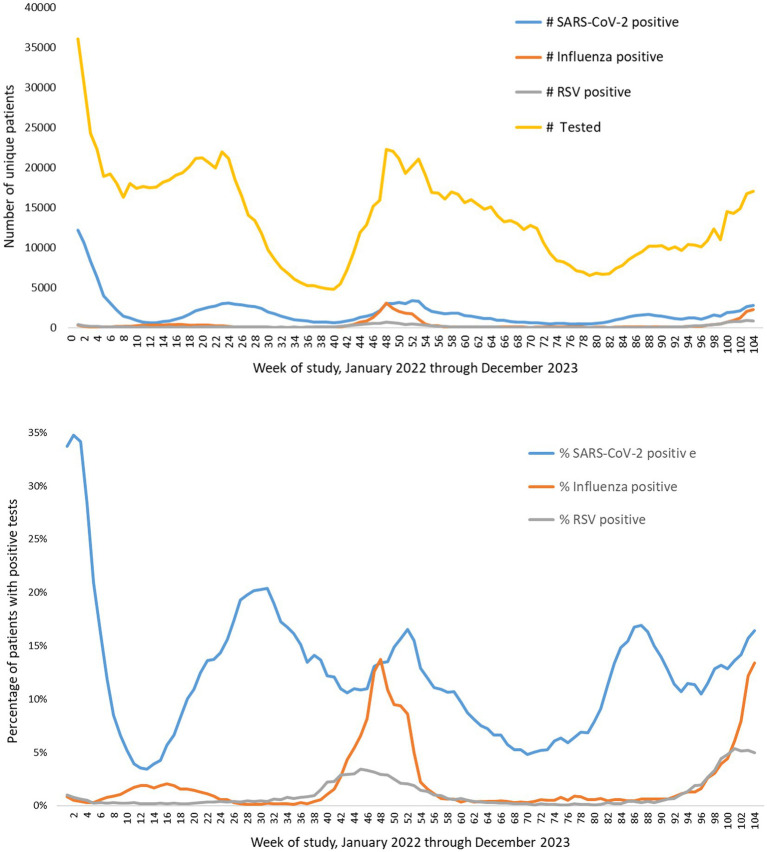
Triple-testing over time. Data are plotted by week, starting at January 1, 2022, and ending at December 31, 2023. Top panel: numbers of unique patients tested the same week for SARS-CoV-2, influenza, and RSV, and the numbers who tested positive. Bottom panel: percentages of unique tested patients who tested positive for SARS-CoV-2, influenza, or RSV.

From January 1, 2022 to December 31, 2023, 835,987 Veteran patients underwent same-week testing for SARS-CoV-2, influenza, and RSV (“triple-tested”). Among these, 170,592 (20.4%) were positive for SARS-CoV-2 alone, 30,454 (3.6%) for influenza alone, 13,207 (1.6%) for RSV alone, 1,300 (0.2%) for more than one of these viruses, and 620,434 (74.2%) were negative for all three. Among the 1,300 patients with multiple viruses detected, 742 (57.1%) were positive for SARS-CoV-2 and influenza, 425 (32.7%) for SARS-CoV-2 and RSV, 131 (10.1%) for influenza and RSV, and 2 (0.02%) for all 3 viruses The total numbers of patients tested, numbers of positive tests, and percentage of tests positive over time are shown in Figure 1. The percentage of patients positive for SARS-Cov-2 was high (peak of about 30%) in early 2022 and very low for influenza and RSV, coinciding with the first omicron wave. After that, positive testing for influenza and RSV corresponded to the usual seasonal pattern in the winter of 2022–23, whereas SARS-CoV-2 included a peak that winter but also at other times before and after.

Demographic and clinical characteristics of the subcohorts defined by infection type are shown in Table 1 and Supplementary Tables 2 and 3. Patients with influenza were younger (mean age 57.7, versus 62.2 for SARS-CoV-2, 63.3 for RSV, and 60.1 for multiple viruses) and had lower proportions with pre-existing cardiovascular diseases (atrial fibrillation, heart failure, ischemic heart disease, peripheral vascular disease, and stroke) and other age-related comorbidities (e.g., Alzheimer’s/dementia, prostate cancer). Characteristics were otherwise similar across groups—including between patients with multiple positive tests and those with a single infection.

**Table 1:** Key demographic and clinical characteristics of the subcohorts, defined by results of tests for viral infections.

	All negative	SARS-CoV-2 only	Influenza only	RSV only	Multiple positive
Total, *N* (%)	620,434	170,592	30,454	13,207	1,300
Age ranges, *N* (%)					
<50	130,702 (21.2)	39,436 (23.1)	9,681 (31.8)	2,587 (19.6)	342 (26.3)
50 to <60	101,201 (16.3)	30,246 (17.7)	5,748 (18.9)	2,284 (17.3)	237 (18.2)
60 to <70	147,364 (23.8)	36,878 (21.6)	6,818 (22.4)	2,998 (22.7)	296 (22.8)
70 to <80	181,184 (29.2)	46,752 (27.4)	6,494 (21.3)	4,029 (30.5)	331 (25.5)
≥80	59,909 (9.7)	17,280 (10.1)	1,713 (5.6)	1,309 (9.9)	94 (7.2)
Unknown	74 (0.0)	21 (0.0)	3 (0.0)	2 (0.0)	0 (0.0)
Sex, *N* (%)					
F	68,863 (11.1)	20,251 (11.9)	4,089 (13.4)	1,706 (12.9)	160 (12.3)
M	551,497 (88.9)	150,321 (88.1)	26,362 (86.6)	11,500 (87.1)	1,140 (87.7)
Unknown	74 (0.0)	20 (0.0)	3 (0.0)	1 (0.0)	0 (0.0)
Vaccinated (SARS-CoV-2), *N* (%)	498,913 (80.4)	132,240 (77.5)	23,400 (76.8)	10,955 (82.9)	1,038 (79.8)
BMI class extremes, *N* (%)					
Severe obesity	52,222 (8.4)	14,983 (8.8)	2,969 (9.7)	1,264 (9.6)	126 (9.7)
Underweight	7,890 (1.3)	1,698 (1.0)	211 (0.7)	128 (1.0)	8 (0.6)
Comorbidities, *N* (%)					
Alzheimer/dementia	40,523 (6.5)	12,717 (7.5)	1,198 (3.9)	874 (6.6)	76 (5.8)
Anemia	99,474 (16.0)	28,520 (16.7)	3,805 (12.5)	2,198 (16.6)	176 (13.5)
Cancer	72,634 (11.7)	18,754 (11.0)	2,612 (8.6)	1,579 (12.0)	144 (11.1)
Cardiovascular disease	446,759 (72.0)	122,539 (71.8)	20,050 (65.8)	9,936 (75.2)	904 (69.5)
Chronic kidney disease	119,083 (19.2)	35,232 (20.7)	4,867 (16.0)	2,822 (21.4)	218 (16.8)
COPD/bronchiectasis	102,000 (16.4)	25,719 (15.1)	4,719 (15.5)	2,491 (18.9)	211 (16.2)
Diabetes	196,016 (31.6)	53,990 (31.6)	8,269 (27.2)	4,297 (32.5)	371 (28.5)
Immune-suppressive drugs	97,848 (15.8)	27,231 (16.0)	6,819 (22.4)	4,521 (34.2)	309 (23.2)
Impaired function/mobility	33,188 (5.3)	9,095 (5.3)	976 (3.2)	605 (4.6)	54 (4.2)
Liver disease	56,054 (9.0)	15,137 (8.9)	2,517 (8.3)	1,147 (8.7)	118 (9.1)
Neurologic disease	110,882 (17.9)	31,508 (18.5)	4,608 (15.1)	2,550 (19.3)	216 (16.6)
Psychiatric condition	293,371 (47.3)	80,500 (47.2)	14,853 (48.8)	6,271 (47.5)	600 (46.2)
Tobacco use	121,542 (19.6)	28,094 (16.5)	5,761 (18.9)	2,119 (16.0)	220 (16.9)
Vascular disease	158,008 (25.5)	42,628 (25.0)	5,993 (19.7)	3,441 (26.1)	284 (21.8)

BMI, body mass index; COPD, chronic obstructive pulmonary disease.

Numbers in parentheses indicate the percentages of patients in the column who had that characteristic. See Supplementary Table 1 for definitions and Supplementary Tables 2 and 3 for additional data.

Hypoxemia was experienced by 8.0% of patients with SARS-CoV-2 alone, 7.7% with influenza alone, 9.3% with RSV alone, 8.7% with more than one virus, and 8.9% with only negative tests (Table 2; Supplementary Table 4). Death occurred in 1.9% of patients with SARS-CoV-2 alone, 0.8% with influenza alone, 1.1% with RSV alone, 1.5% with more than one virus, and 2.0% with only negative tests (Table 3; Supplementary Table 5). Death was preceded by evidence of hypoxemia in similar percentages of cases (58.1% SARS-CoV-2, 58.2% influenza, 60.8% RSV, 68.4% multiple viruses, *p* = 0.49) and occurred in 1.1% of all patients with SARS-CoV-2 alone, 0.4% with influenza alone, 0.7% with RSV alone, and 1.2% with multiple viruses. Hypoxemia (7.5% versus 8.0%) and death (1.6% versus 1.9%) were less frequent for patients with SARS-CoV-2 infection when excluding cases before April, 2022 (which featured many SARS-CoV-2 cases and few influenza or RSV cases), but these data indicate that the higher mortality after SARS-CoV-2 than influenza or RSV was not driven entirely by the first omicron wave. In addition, similar reductions in hypoxemia and death were seen among patients who tested negative for all three viruses when excluding cases before April, 2022 (unpublished data), suggesting that reduced mortality in any group after March, 2022, may not have been related to viral infection.

**Table 2:** Hypoxemia among patients with different test results for viral infection.

Virus test result	All negative		SARS-CoV-2 only		Influenza only		RSV only		Multiple positive	
Hypoxemia	No	Yes	No	Yes	No	Yes	No	Yes	No	Yes
Total, *N* (%)	565,299	55,135 (8.9)	156,924	13,668 (8.0)	28,104	2,350 (7.7)	11,985	1,222 (9.3)	1,187	113 (8.7)
Age ranges										
<50	128,368	2,334 (1.8)	38,999	416 (1.1)	9,591	87 (0.9)	2,550	35 (1.4)	340	2 (0.6)
50 to <60	96,569	4,632 (4.6)	29,308	938 (3.1)	5,547	201 (3.5)	2,197	87 (3.8)	229	8 (3.4)
60 to <70	133,658	13,706 (9.3)	33,970	2,908 (7.9)	6,136	682 (10.0)	2,693	305 (10.2)	271	25 (8.4)
70 to <80	157,404	23,780 (13.1)	40,687	6,065 (13.0)	5,468	1,026 (15.8)	3,478	551 (13.7)	275	56 (16.9)
≥80	49,226	10,683 (17.8)	13,939	3,341 (19.3)	1,359	354 (20.7)	1,065	244 (18.6)	72	22 (23.4)
Sex										
F	65,888	2,975 (4.3)	19,694	557 (2.8)	3,924	165 (4.0)	1,623	83 (4.9)	153	7 (4.4)
M	499,337	52,160 (9.5)	137,210	13,111 (8.7)	24,177	2,185 (8.3)	10,361	1,139 (9.9)	1,034	106 (9.3)
Vaccinated (SARS-CoV-2)	452,520	46,393 (9.3)	121,945	10,295 (7.8)	21,405	1,995 (8.5)	9,899	1,056 (9.6)	946	92 (8.9)
BMI class extremes										
Severe obesity	46,608	5,614 (10.8)	13,715	1,268 (8.5)	2,732	237 (8.0)	1,123	141 (11.2)	116	10 (7.9)
Underweight	5,989	1,901 (24.1)	1,228	470 (27.7)	152	59 (28.0)	80	48 (37.5)	3	5 (62.5)
Comorbidities										
Alzheimer/dementia	32,723	7,800 (19.2)	9,584	3,133 (24.6)	874	324 (27.0)	640	234 (26.8)	59	17 (22.4)
Anemia	84,296	15,178 (15.3)	23,690	4,830 (16.9)	3,147	658 (17.3)	1,798	400 (18.2)	141	35 (19.9)
Cancer	62,595	10,039 (13.8)	16,064	2,690 (14.3)	2,231	381 (14.6)	1,331	248 (15.7)	119	25 (17.4)
Cardiovascular disease	400,510	46,249 (10.4)	110,637	11,902 (9.7)	18,037	2,013 (10.0)	8,837	1,099 (11.1)	802	102 (11.3)
Chronic kidney disease	101,711	17,372 (14.6)	29,671	5,561 (15.8)	4,102	765 (15.7)	2,332	490 (17.4)	177	41 (18.8)
COPD/bronchiectasis	79,929	22,071 (21.6)	20,113	5,606 (21.8)	3,523	1,196 (25.3)	1,846	645 (25.9)	154	57 (27.0)
Diabetes	172,941	23,075 (11.8)	47,728	6,262 (11.6)	7,300	969 (11.7)	3,730	567 (13.2)	309	62 (16.7)
Immune-suppressive drugs	82,073	15,775 (16.1)	23,028	4,203 (15.4)	5,623	1,196 (17.5)	3,785	736 (16.3)	259	50 (16.2)
Impaired function/mobility	26,798	6,390 (19.3)	6,982	2,113 (23.2)	736	240 (24.6)	461	144 (23.8)	40	14 (25.9)
Liver disease	50,143	5,911 (10.5)	13,496	1,641 (10.8)	2,244	273 (10.8)	994	153 (13.3)	104	14 (11.9)
Neurologic disease	97,577	13,305 (12.0)	27,572	3,936 (12.5)	4,019	589 (12.8)	2,179	371 (14.5)	187	29 (13.4)
Psychiatric condition	270,338	23,033 (7.9)	74,375	6,125 (7.6)	13,803	1,050 (7.1)	5,724	547 (8.7)	549	51 (8.5)
Tobacco use	107,301	14,241 (11.7)	24,860	3,234 (11.5)	4,975	786 (13.6)	1,780	339 (16.0)	185	35 (15.9)
Vascular disease	135,155	22,853 (14.5)	36,222	6,406 (15.0)	4,977	1,016 (17.0)	2,871	570 (16.6)	236	48 (16.9)

BMI, body mass index; COPD, chronic obstructive pulmonary disease.

Numbers in parentheses indicate the percentages of patients with that variable/characteristic who experienced hypoxemia within 14 days of the index date. See Supplementary Table 4 for additional data.

**Table 3:** Death among patients with different test results for viral infection.

Virus test result	All negative		SARS-CoV-2 only		Influenza only		RSV only		Multiple positive	
Death	No	Yes	No	Yes	No	Yes	No	Yes	No	Yes
Totals, *N* (%)	607,906	12,528 (2.0)	167,429	3,163 (1.9)	30,222	232 (0.8)	13,059	148 (1.1)	1,281	19 (1.5)
Age ranges										
<50	130,481	221 (0.2)	39,384	31 (0.1)	9,676	2 (0.0)	2,585	0 (0.0)	342	0 (0.0)
50 to <60	100,641	560 (0.6)	30,128	118 (0.4)	5,732	16 (0.3)	2,279	5 (0.2)	237	0 (0.0)
60 to <70	145,066	2,298 (1.6)	36,387	491 (1.3)	6,782	36 (0.5)	2,976	22 (0.7)	292	4 (1.4)
70 to <80	175,985	5,199 (2.9)	45,438	1,314 (2.8)	6,398	96 (1.5)	3,966	63 (1.6)	322	9 (2.7)
≥80	556,59	4,250 (7.1)	16,071	1,209 (7.0)	1,631	82 (4.8)	1,251	58 (4.4)	88	6 (6.4)
Sex										
F	68,509	354 (0.5)	20,186	65 (0.3)	4,081	8 (0.2)	1,701	5 (0.3)	160	0 (0.0)
M	539,323	12,174 (2.2)	147,223	3,098 (2.1)	26,138	224 (0.8)	11,357	143 (1.2)	1,121	19 (1.7)
Vaccinated (SARS-CoV-2)	488,988	9,925 (2.0)	130,070	2,170 (1.6)	23,205	195 (0.8)	10,828	127 (1.2)	1,024	14 (1.3)
BMI class extremes										
Severe obesity	51,720	502 (1.0)	14,839	144 (1.0)	2,964	5 (0.2)	1,256	8 (0.6)	125	1 (0.8)
Underweight	6,948	942 (11.9)	1,488	210 (12.4)	196	15 (7.1)	109	19 (14.8)	8	0 (0.0)
Comorbidities										
Alzheimer/dementia	37,578	2,945 (7.3)	11,711	1,006 (7.9)	1,131	67 (5.6)	822	52 (5.9)	70	6 (7.9)
Anemia	94,898	4,576 (4.6)	27,084	1,436 (5.0)	3,705	100 (2.6)	2,122	76 (3.5)	166	10 (5.7)
Cancer	69,199	3,435 (4.7)	17,908	846 (4.5)	2,557	55 (2.1)	1,538	41 (2.6)	139	5 (3.5)
Cardiovascular disease	436,334	10,425 (2.3)	119,759	2,780 (2.3)	19,844	206 (1.0)	9,799	137 (1.4)	887	17 (1.9)
Chronic kidney disease	114,423	4,660 (3.9)	33,718	1,514 (4.3)	4,764	103 (2.1)	2,734	88 (3.1)	209	9 (4.1)
COPD/bronchiectasis	98,119	3,881 (3.8)	24,669	1,050 (4.1)	4,621	98 (2.1)	2,425	66 (2.6)	204	7 (3.3)
Diabetes	191,048	4,968 (2.5)	52,565	1,425 (2.6)	8,169	100 (1.2)	4,224	73 (1.7)	360	11 (3.0)
Immune-suppressive drugs	95,228	2,620 (2.7)	26,374	857 (3.1)	6,744	75 (1.1)	4,460	61 (1.3)	301	8 (2.6)
Impaired function/mobility	31,376	1,812 (5.5)	8,477	618 (6.8)	949	27 (2.8)	566	39 (6.4)	50	4 (7.4)
Liver disease	54,340	1,714 (3.1)	14,647	490 (3.2)	2,479	38 (1.5)	1,123	24 (2.1)	115	3 (2.5)
Neurologic disease	108,113	2,769 (2.5)	30,640	868 (2.8)	4,548	60 (1.3)	2,513	37 (1.5)	211	5 (2.3)
Psychiatric condition	289,037	4,334 (1.5)	79,272	1,228 (1.5)	14,754	99 (0.7)	6,206	65 (1.0)	591	9 (1.5)
Tobacco use	118,797	2,745 (2.3)	27,442	652 (2.3)	5,703	58 (1.0)	2,092	27 (1.3)	216	4 (1.8)
Vascular disease	152,386	5,622 (3.6)	40,992	1,636 (3.8)	5,859	134 (2.2)	3,355	86 (2.5)	275	9 (3.2)

BMI, body mass index; COPD, chronic obstructive pulmonary disease.

Numbers in parentheses indicate the percentages of patients with that variable/characteristic who died within 4 weeks of the index date. See Supplementary Table 5 for additional data.

Independent of the presence or type of viral infection, patients with age > 70 or a wide range of comorbidities had higher incidences of hypoxemia and/or death (Tables 2, 3; Supplementary Tables 4 and 5).

The primary analyses, comparing the odds of hypoxemia or the odds of death after the different viral infections, were limited to patients in whom at least one test for infection was positive. Among many variables known to be associated with severe outcomes of SARS-CoV-2 infection, only age and receipt of immune-suppressive medications met the definition of being confounders (see Methods) of the association between the infection type and these outcomes. After adjustment for age and receipt of immune-suppressive drugs, several small but statistically significant associations between hypoxemia and infection type were noted (Table 4): compared to SARS-CoV-2 infection alone, hypoxemia was more common for influenza alone (aOR 1.12, 95% CI 1.06–1.17; aRD 0.9%), lower for RSV alone (aOR 0.91, 95% CI 0.85–0.97; aRD–0.7%), and not significantly different for multiple infections (aOR 1.09, 95% CI 0.89–1.34; aRD 0.7%). Results were similar when covariates were chosen *a priori* and included in the full model if significantly associated individually (Table 4): aOR for hypoxemia 1.17 (1.11–1.23) with influenza, 0.96 (0.90–1.03) with RSV, and 1.13 (0.92–1.40) with multiple infections. aOR for the covariates are shown in Supplementary Table 6 (all infections in one model) and 7 (stratified by infection). The aOR were attenuated by inclusion in a single multivariable model but remained significant for age and 12 or the 15 other selected variables.

**Table 4:** Odds ratios (OR) and risk differences for association between hypoxemia (within 14 days) or death (within 4 weeks) and a positive test result for influenza, RSV, or more than one of these viruses, relative to SARS-CoV-2, with or without adjustment for age and use of immune-suppressive drugs (columns 4–5) or additional clinical variables including comorbidities (columns 6–7).

Positive test result	Outcome *N* = 215,553	OR, unadjusted (95% CI)	OR, adjusted^*^ (95% CI)	Risk difference, adjusted^*^ (95% CI)	OR, adjusted^**^ (95% CI)	Risk difference, adjusted^**^ (95% CI)
	Hypoxemia yes/total 17,353/215,553 (8.1%)					
Influenza only (*n* = 30,454)	2,350/30,454 (7.7%)	0.96 (0.92–1.01)	1.12 (1.06–1.17)	0.9% (0.4–1.2)	1.17 (1.11–1.23)	1.2% (0.8–1.7)
RSV only (*n* = 13,207)	1,222/13,207 (9.3%)	1.17 (1.10–1.25)	0.91 (0.85–0.97)	−0.7% (−1.1 to −0.2)	0.96 (0.90–1.03)	−0.3% (−0.7 to 0.2)
Multiple (*n* = 1,300)	113/1,300 (8.7%)	1.09 (0.90–1.33)	1.09 (0.89–1.34)	0.7% (−0.8 to 2.4)	1.13 (0.92–1.40)	1.0% (−0.6 to 2.9)
SARS-CoV-2 only (*n* = 170,592)	13,688/170,592 (8.0%)	Ref (1.00)	Ref (1.00)	Ref	Ref	Ref
	Died yes/total 3,562/215,553 (1.7%)					
Influenza only (*n* = 30,454)	232/30,454 (0.8%)	0.41 (0.36–0.47)	0.52 (0.46–0.60)	−0.9% (−1.0 to −0.7)	0.59 (0.51–0.67)	−0.8% (−0.9 to −0.6)
RSV only (*n* = 13,207)	148/13,207 (1.1%)	0.60 (0.51–0.71)	0.51 (0.43–0.60)	−0.9% (−1.0 to −0.7)	0.55 (0.46–0.65)	−0.8% (−1.0 to −0.6)
Multiple positive (*n* = 1,300)	19/1,300 (1.5%)	0.79 (0.50–1.24)	0.86 (0.54–1.36)	−0.3% (−0.8 to 0.7)	0.92 (0.58–1.47)	−0.1% (−0.8 to 0.8)
SARS-CoV-2 only (*n* = 170,592)	3,163/170,592 (1.9%)	Ref (1.00)	Ref (1.00)	Ref	Ref	Ref

* Age was included in the model as a 3-part spline and use of immune-suppressive drugs included as a dichotomous (yes/no) variable.

** Age was included as a 3-part spline, and 15 additional clinical and demographic variables were included as dichotomous variables.

Inclusion of interaction terms showed a significant interaction among influenza infection, use of immune-suppressive drugs, and age. An analysis stratified by use of immune-suppressive drugs gave a paradoxical result: the odds of hypoxemia with influenza were higher than with SARS-CoV-2 among patients taking immune-suppressive drugs (aOR 1.38, 95% CI 1.28–1.49; aRD 4.7%), but not among patients not taking immune-suppressive drugs (aOR 0.95, 95% CI 0.90–1.02; aRD–0.3%). Similarly, the odds of hypoxemia with RSV were similar to SARS-CoV-2 among patients taking immune-suppressive drugs (aOR 1.04, 95% CI 0.95–1.13; aRD 0.5%) but lower among patients not taking such drugs (aOR 0.81, 95% CI 0.73–0.89; aRD–1.2%) (Table 5).

**Table 5:** Age-adjusted odds ratios (OR) and risk differences for association between hypoxemia (within 14 days) or death (within 4 weeks) and a positive test result for influenza, RSV, or more than one of these viruses, relative to SARS-CoV-2.

Patients taking immune-suppressive medications				Patients not taking immune-suppressive medications		
Positive test result (*N* = all patients)	Outcome	OR, age-adjusted (95% CI)	Risk difference, age-adjusted (95% CI)	Outcome	OR, age-adjusted (95% CI)	Risk difference, age adjusted (95% CI)
	Hypoxemia yes/total (%) 6,185/38,880 (15.9%)			Hypoxemia yes/total (%) 11,168/176,673 (6.3%)		
Influenza only (*n* = 30,454)	1,196/6,819 (17.5%)	1.38 (1.28–1.49)	4.7% (3.5–6.0)	1,154/23,635 (4.9%)	0.95 (0.90–1.02)	−0.3% (−0.6 to 0.1)
RSV only (*n* = 13,207)	736/4,521 (16.3%)	1.04 (0.95, 1.13)	0.5% (−0.7 to 1.7)	486/8,686 (5.6%)	0.81 (0.73–0.89)	−1.2% (−1.7 to −0.7)
Multiple (*n* = 1,300)	50/309 (16.2%)	1.08 (0.79, 1.47)	1.0% (−2.8 to 5.7)	63/991 (6.4%)	1.12 (0.86–1.46)	0.7% (−0.9 to 2.8)
SARS-CoV-2 Only (*n* = 170,592)	4,203/27,231 (15.4%)	Ref (1.00)	Ref	9,465/143,361 (6.6%)	Ref (1.00)	Ref
	Died Yes/total (%) 1,001/38,880 (2.6%)			Died Yes/total (%) 2,561/176,673 (1.5%)		
Influenza only (*n* = 30,454)	75/6,819 (1.1%)	0.41 (0.32, 0.52)	−1.8% (−2.1 to −1.5)	157/23,635 (0.7%)	0.59 (0.50–0.69)	−0.7% (−0.8 to −0.5)
RSV only (*n* = 13,207)	61/4,521 (1.4%)	0.41 (0.31, 0.53)	−1.8% (−2.2 to −1.5)	87/8,686 (1.0%)	0.60 (0.48–0.74)	−0.6% (−0.8 to −0.4)
Multiple positive (*n* = 1,300)	8/309 (2.6%)	0.85 (0.42, 1.73)	−0.5% (−1.8 to 2.2)	11/991 (1.1%)	0.85 (0.46–1.55)	−0.2% (−0.9 to 0.9)
SARS-CoV-2 only (*n* = 170,592)	857/27,231 (3.2%)	Ref (1.00)	Ref	2,306/143,361 (1.6%)	Ref (1.00)	Ref

Separate analyses were patients taking (columns 2–4) or not taking (columns 5–7) immune-suppressive drugs. Age was modeled as a 3-part spline.

Analyses using death within 4 weeks as the outcome gave similar associations with specific comorbidities, with or without adjustment (Supplementary Tables 6–8). In this multivariable approach, the odds of death were lower with influenza alone (aOR 0.59, CI 0.51–0.67; aRD–0.8%) or RSV alone (aOR 0.55, CI 0.46–0.65; aRD–0.8%) than with SARS-CoV-2 alone, with no difference seen for multiple infections (aOR 0.92, CI 0.58–1.47; aRD–0.1%). Again, only age and use of immune-suppressive drugs met criteria as confounders, and results of multivariable modeling with this parsimonious approach gave similar results: aOR for death 0.52 (CI 0.46–0.60) with influenza, aOR 0.51 (CI 0.43–0.60) with RSV, and aOR 0.86 (CI 0.54–1.36) with multiple infections (Table 4). In contrast to the analysis of hypoxemia, no interaction terms were statistically significant, and odds of death did not differ in subgroups stratified by use or non-use of immune-suppressive drugs. Thus, SARS-CoV-2 was associated with significantly higher short-term mortality but not with a higher incidence of hypoxemia.

## Discussion

High prevalence of multiple respiratory viruses simultaneously has the potential to overwhelm health systems. Additionally, patients infected with multiple respiratory viruses concurrently might be more likely to have severe disease than those infected with only one virus. In this large national cohort observed over multiple years, we found that co-infection was rare, comprising 0.2% of triple-tested patients and 0.6% of patients where at least virus was detected. Co-infected patients did not appear to have more severe disease than patients infected with one virus, regardless of immune status, but the numbers of cases were too small to rule out modest-sized differences, especially for mortality.

The small number of population-based studies, whether in 2020 or later, also found co-infection to be rare. A lab-based study of samples collected 2019–2021 reported no detection of another respiratory pathogen among 29,000 samples positive for SARS-CoV-2 (13). Another lab-based study reported, among 1.3 million tests, simultaneous positive testing for SARS-CoV-2 and influenza in 0.1%, for SARS-CoV-2 and RSV in 0.03%, for influenza and RSV in 0.07%, and for all three in 0.001%. The total percentages of tests positive for individual viruses was not provided, but a graph suggested 10–20% positive for at least one, indicating that the incidences of co-infections among all positive tests was similar to what we found (14). Finally, and of particular interest for our study, a lab-based study using nationwide VA data from 2020 reported that among 3,757 patients positive for SARS-CoV-2 who were tested for other respiratory pathogens, 56 (1.5%) were positive for something, and of those, only 15 were positive for influenza and 4 for RSV (15). These data indicate that when data are not limited to hospitalized or critically ill patients, co-infection with influenza or RSV is not only rare in the post-vaccination era (our study) but also was in 2020 (published studies). Numerous reports of higher incidences in 2020 were often limited to hospitalized or critically ill patients (5), and higher incidences were reported from Asia (often >10%) than from the Americas (typically 0–6%) (16).

Our study also adds to the limited literature on outcomes among co-infected patients after 2020, as almost all data on co-infection, even if published in 2021–2023 and reviewed 2023–2025, originate from 2020 data. In a systematic review of over 120 case series and reports, co-infection with SARS-CoV-2 and other respiratory viruses, particularly influenza, was associated with higher risk of more severe respiratory disease during this early period in the pandemic (5). Two meta-analyses of data from 2020 (each with only 12 studies meeting inclusion criteria) arrived at opposite conclusions regarding risk of severe illness (worse versus not different) but agreed that mortality was not increased (6, 17).

The aforementioned VA study (2020 data) is also one of few population-based studies in which outcomes were compared between patients with multiple or single infections. Odds of hospitalization (OR 1.6, CI 0.9–2.8) or death (OR 1.4, CI 0.7–2.7) among patients with SARS-CoV-2 and another respiratory infection were not higher compared to infection with SARS-CoV-2 alone but were higher (for hospitalization, OR 2.8, 95%CI 1.6–5.1; for death, OR 2.8, 95%CI 1.3–5.7) for SARS-CoV-2 co-infection compared to non-SARS-CoV-2 respiratory pathogen mono-infection (*N* = 1022, 23% influenza) (15). Since 2020, we are aware of only one analysis of co-infection in adults. Vieceli, in a study during the first omicron wave in Brazil (November 2021 through March 2022), analyzed data from 92,777 patients with SARS-CoV-2/COVID-19 alone, 11,570 with influenza, and 655 with both (0.6% of the positive tests) (8). Consistent with our findings, patients co-infected had similar odds for ICU admission (aOR 0.88, 95%CI 0.7–1.1) and in-hospital mortality (aOR 1.02, 95%CI 0.84–1.23) as patients with SARS-CoV-2 alone, and patients with influenza alone had lower odds. Thus, although marked declines in absolute rates of hospitalization and death after SARS-CoV-2 infection have been widely reported, it remains unclear that co-infection has ever been associated with increased risk when all patients with documented infections are included.

Studies from multiple countries using data from 2020 and earlier consistently found higher mortality following COVID-19 than after influenza or RSV (18–23). Although several studies after SARS-CoV-2 vaccination became available have found that mortality remained slightly higher after SARS-CoV-2 infection than influenza or RSV (2–4), other studies have found mortality to be at least as high or higher with RSV (24–26). These studies and others (27, 28) have usually concluded that severe non-fatal outcomes (hypoxemia or ICU admission) were higher among unvaccinated patients with RSV than among vaccinated patients with SARS-CoV-2 or influenza. Our results are at odds with these reports but are very similar to those reported by Bajema et al. using a similar dataset and a target trial emulation design (29). We suspect but cannot prove that the study design—inclusion of all patients testing positive during a period when testing was performed both on outpatients with respiratory symptoms and on inpatients regardless of respiratory symptoms—is the source of the different results. The studies referenced above were usually limited to hospitalized patients and/or respiratory illness.

Although odds of hypoxemia and death were 2-3-times higher in patients with cancer, cardiovascular, pulmonary, or neurologic disease, these comorbidities were associated with severe outcomes across all types of infection—or with no viral infection. Incidences of hypoxemia (8.9%) and death (2.0%) after testing, prevalences of comorbidities, and the associations (aOR) of those comorbidities with hypoxemia or death were similar among patients who tested negative for all 3 viruses as among patients with SARS-CoV-2. Although formal comparison to the group of patients who tested negative for all three viruses on triple-testing might seem interesting in principle, the differences in baseline characteristics, including unmeasurable variables, are likely to be so large that residual confounding would be inevitable. These patients are much more likely to have been screened in the absence of symptoms, for reasons varying from elective surgery to admission for life-threatening comorbidities.

With multivariable modeling, aORs for individual comorbidities usually remained statistically significant but dropped below 2. Since several of these comorbidities are expected to be collinear with each other and especially with age, we caution against using these results to compare the impact of these comorbidities to each other. Results of modeling within-group have the separate limitation of being imprecise when the number of events is low, e.g., for analysis of death among patients with multiple infections.

Two important limitations of this study are incomplete data on vaccination and lack of information about whether patients were symptomatic at the time of testing. The fact that SARS-CoV-2 vaccination was not a confounder in bivariable analyses, i.e., was not associated specifically with reduced severity of SARS-CoV-2 compared to influenza or RSV, illustrates the challenge of trying to incorporate documented vaccination status. We suspect that absence of a recorded influenza vaccine would have a poor negative predictive value, since vaccines are given seasonally and are widely available at non-VA pharmacies and clinics. A predictor variable for “influenza vaccination” might instead serve as a surrogate for the degree of use of VA versus non-VA care, which would be undesirable. In any case, Bajema et al. included influenza vaccination (documented for 28% of Veterans) in their analysis but primarily to compare outcomes for COVID-19 and influenza stratified by vaccination status, with the finding that outcomes were worse for COVID-19 than influenza among unvaccinated but not among vaccinated patients (29). Finally, RSV vaccination was not available until mid-2023, as reflected in a reported vaccination status of 0% in the 2022–23 season and 2.2% in the 2023–2024 season (29). Therefore, our results may not be generalizable to populations with higher influenza or RSV coverage.

Widespread use of triple-test screening independent of symptoms has several implications. First, it is anticipated that the incidence of hypoxemia would be lower than in cohorts in which screening was symptom-based. This may well explain the lower rate of hypoxemia in older adults with RSV infection than has been reported in some other studies. However, limiting our dataset to patients tested for all 3 viruses on the same day provides reassurance that comparison of different groups within the dataset is not confounded by indication. Second, the disconnect between the increased incidences of hypoxemia and death with SARS-CoV-2 versus influenza or RSV (similar risk differences of ~1% for both outcomes but with high aOR for death and minimal aOR for hypoxemia) suggests that although the numbers of patients with hypoxemia is similar, the risk of respiratory failure remains somewhat higher with SARS-CoV-2. The preserved small difference in mortality after counting only patients with hypoxemia before death supports this tentative conclusion.

This study has several additional limitations. First, results may not be generalizable to women, children, or adults of Asian, Hispanic/Latino, or Native American ethnicity. Second, the effect of previous infection with a similar or different virus was not assessed, because attempting to do so would have added analytic complexity and would have increased the importance of missing data. Therefore, only the first infection detected on triple-testing in 2022–23 was analyzed. In order to retain more patients with influenza or RSV, a secondary analysis was done with the start time of April 1, 2022, after the first and largest wave of SARS-CoV-2 omicron cases in 2022 and before the re-emergence of influenza and RSV in their usual seasonal pattern (unpublished data). Third, it is likely that some positive tests reflected infections that had resolved clinically. Using only the first positive test mitigates this concern somewhat, but the widespread use of home testing for SARS-CoV-2 and the frequently mild presentations of all 3 of these infections means that the calculated incidences of active infection are inflated. This limitation is intrinsic to study of this question in a large database, since attempting to identify and limit to active infections would involve arbitrary decisions and undermine the research question, in which inclusion of clinically insignificant infection was desirable. Another, related limitation is that the different testing modalities may not have the same sensitivity, especially as relates to antigen versus PCR testing but also for any modality among many manufacturers. The great majority of tests were PCR, since we excluded all tests labeled POC (point of care) and testing on hospital admission, a policy driven by infection-control concerns, typically uses PCR because of its greater sensitivity and the lack of a need for immediate results.

Fourth, causes of death could not readily be determined. In our previous work, by mid-2022, COVID-19 caused or contributed to death in about one-half of VA patients who died within 30 days of infection (30, 31), but we are not aware of similar studies among patients who died after influenza or RSV. Considering that the rates of hypoxemia before death did not differ significantly among the infection groups (SARS-CoV-2, influenza, RSV, or multiple infections) in this study, as well as the similar demographic and clinical characteristics of these groups, we see no reason to propose that deaths for reasons unrelated to respiratory illness or unrelated to infection at all were more common among patients with SARS-CoV-2 than with influenza or RSV, although a greater role for non-respiratory disease with SARS-CoV-2 would have been a plausible finding. Since mortality without evidence of prior hypoxemia could occur for multiple reasons not amenable to analysis of structured data, including incidental infection or hospital care outside VA, we did not feel that investigation of causes of death absent hypoxemia would be fruitful.

## Conclusion

In a cohort of patients (outpatient or inpatient) tested the same week for SARS-CoV-2, influenza, and RSV over a 2-year period after the initial COVID-19 pandemic, viral co-infection was rare. Coinfection did not appear to be associated with greater incidence of hypoxemia or death than infection with SARS-CoV-2 alone, although the numbers of cases were too small to rule out modest-sized differences, especially for mortality. Death was less frequent with influenza or RSV than SARS-CoV-2.

## Data Availability

The data analyzed in this study is subject to the following licenses/restrictions: the dataset consists entirely of variables derived from electronic health records of hundreds of thousands of patients. Requests to access these datasets should be directed to Paul.Monach@va.gov.
