# Supplementary material for: Severe outcomes of individual or multiple respiratory viral infections in a large national healthcare system, 2022–2023
Source: Front Public Health. 2026 Apr 22;14:1808528. doi: 10.3389/fpubh.2026.1808528 (PMC13144013; doi:10.3389/fpubh.2026.1808528)
Supplement: Supplementary file 1 [file Data_Sheet_1.DOCX]

**Supplementary Material**

**Burden and Severity of Respiratory Viruses in a Large National Healthcare System: A Retrospective Cohort Study 2022-2023**

Janet M. Grubber, Ikwo K. Oboho, Kaitlin N. Swinnerton, Theodore C. Feldman, Nhan V. Do, Nathanael R. Fillmore, Westyn Branch-Elliman, Paul A. Monach

**Contents**

sTable 1. Data dictionary 2

sTable 2. Demographic and clinical characteristics of the cohort, stratified by test results for viral infections 6

sTable 3. Demographic and clinical characteristics of patients with multiple viral infections 9

sTable 4. Hypoxemia among patients with different test results for viral infections 12

sTable 5. Death among patients with different test results for viral infections 15

sTable 6. Associations of selected clinical features with outcomes of hypoxemia or death, with and without adjustment for age 18

sTable 7. Associations of selected clinical features with the outcome of hypoxemia, stratified by type of viral infection 19

sTable 8. Associations of selected clinical features with the outcome of death, stratified by type of viral infection 20

**sTable 1. Data dictionary**

| Variable | Definition |
| --- | --- |
| Positive test for viral infection | Positive test for SARS-CoV-2, influenza, and/or RSV, by PCR or antigen testing. Only triple-tested patients were included. For patients with a positive test, only the positive test(s) identified during the first triple-tested week with a positive test were used. For patients with only negative tests during all triple-tested weeks, the date of the first negative test in the first triple-tested week was used. |
| Triple-tested | Testing for SARS-CoV-2, influenza, and RSV during the same week. All analyses were limited to this dataset. |
| Index date | For a triple-tested week chosen to include in analyses as above, the date of the first test performed that week (for any virus, positive or negative) was made the index date. |
| Hypoxemia | Hospitalized with SpO2 < 90% or use of supplemental oxygen at a flow rate > 2L/min at any time during the first 14 days after the index date. |
| Sex | As recorded in structured data in the VA EHR, self-reported by patients at enrollment in VA care. At the time when most patients enrolled, additional questions about gender were not typically recorded. |
| Race | As recorded in structured data in the VA EHR, self-reported by patients at enrollment in VA care. At the time when most patients enrolled, the categories were American Indian or Alaska Native, Asian, Black or African American, Native Hawaiian or Other Pacific Islander, or White, without a category for “other” or mixed/multiple. “Unknown” indicates that no category was selected by the patient. |
| Ethnicity | As recorded in structured data in the VA EHR. Hispanic/Latino ethnicity is self-reported separately from race. “Unknown” indicates that no category was selected by the patient. |
| Age | In years at the index date |
| Age ranges | Age ranges were used for description as below. For adjustment in multivariable logistic regression, age was modeled as a 3-part spline to better model the non-linear association of the outcomes (hypoxemia or death) with age. |
| Less than 40 | Age < 40 |
| 40-45 | Age ≥ 40 AND Age < 45 |
| 45-50 | Age ≥ 45 AND Age < 50 |
| 50-55 | Age ≥ 50 AND Age < 55 |
| 55-60 | Age ≥ 55 AND Age < 60 |
| 60-65 | Age ≥ 60 AND Age < 65 |
| 65-70 | Age ≥ 65 AND Age < 70 |
| 70-75 | Age ≥ 70 AND Age < 75 |
| 75-80 | Age ≥ 75 AND Age < 80 |
| 80 or greater | Age ≥ 80 |
| BMI | Calculated using most recent weight in kg within 5 years, and most recent height ever recorded. If height missing, then used US averages of 1.78m for males and 1.63m for females |
| BMI Class |  |
| Underweight | BMI < 18.5 |
| Normal | BMI ≥ 18.5 AND BMI < 25 |
| Overweight | BMI ≥ 25 AND BMI < 30 |
| Obesity I | BMI ≥ 30 AND BMI < 35 |
| Obesity II | BMI ≥ 35 AND BMI < 40 |
| Severe Obesity | BMI ≥ 40 |
| Unknown | Weight unavailable |
| Region | Geographical region was defined based on the VA facility from which each patient received their full vaccination (second dose of mRNA vaccine or first dose of adenoviral vaccine) |
| Continental | Arkansas, Colorado, Louisiana, Mississippi, Montana, Oklahoma, Texas, Utah, Wyoming |
| Midwest | Illinois, Indiana, Michigan, Minnesota, Missouri, Nebraska, North Dakota, Ohio, South Dakota, Wisconsin |
| North Atlantic | Connecticut, Delaware, District of Columbia, Maine, Maryland, Massachusetts, New Hampshire, New Jersey, New York, North Carolina, Pennsylvania, Rhode Island, Vermont, Virginia, West Virginia |
| Pacific | Alaska, Arizona, California, Hawaii, Idaho, Nevada, New Mexico, Oregon, Washington |
| Southeast | Alabama, Florida, Georgia, Kentucky, Puerto Rico, South Carolina, Tennessee |
| Unknown | Data on the VA facility not available |
| Vaccine Type (SARS-CoV-2) | Defined based on the type of the initial vaccination, as recorded in the VA COVID-19 Shared Data Resource. (Patients with first and second doses of different types were excluded.) |
| Janssen | Patient considered vaccinated 14 days after 1^st^ dose |
| Moderna | Patient considered vaccinated 14 days after 2^nd^ dose |
| Pfizer | Patient considered vaccinated 14 days after 2^nd^ dose |
| None | No record of SARS-CoV-2 vaccination. Will include both truly unvaccinated patients and patients vaccinated outside the VA without vaccination recorded. |
| History of infection before vaccinated | Previous positive SARS-CoV-2 test (PCR or antigen) no later than 14 days after the second mRNA vaccine or first adenoviral vaccine. |
| Cytotoxic Chemotherapy (used as part of definition of Cancer) | Arsenic, asparaginase, asparaginase erwinia chrysanthemi, azacytidine, bendamustine, bleomycin, busulfan, cabazitaxel, calaspargase, capecitabine, carboplatin, carmustine, cedazuridine, chlorambucil, chrysanthemi, cisplatin, cladribine, clofarabine, cyclophosphamide, cytarabine, dacarbazine, dactinomycin, daunorubicin, decitabine, docetaxel, doxorubicin, epirubicin, eribulin, erwinia, etoposide, fludarabine, gemcitabine, hydroxycamptothecin, hydroxyurea, idarubicin, ifosfamide, irinotecan, ixabepilone, lomustine, mechlorethamine, melphalan, mercaptopurine, mitomycin c, mitoxantrone, nelarabine, oxaliplatin, paclitaxel, pegaspargase, pemetrexed, pralatrexate, procarbazine, temozolomide, thioguanine, thiotepa, topotecan, trabectedine, trifluridine, valrubicin, vinblastine, vincristine, vindesine, vinorelbine, within 6 months before breakthrough infection (fluorouracil was excluded because it is also used topically) |
| Immune-suppressive medications before breakthrough infection | When used as a single variable in modeling, includes any of the 4 categories below. Does not include cytotoxic chemotherapy. |
| Cytokine-blocking | Anakinra, benralizumab, brodalumab, canakinumab, certolizumab, dupilumab, etanercept, golimumab, infliximab, ixekizumab, mepolizumab, rilonacept, sarilumab, secukinumab, tocilizumab, ustekinumab, within 3 months before breakthrough infection |
| Glucocorticoids | Methylprednisolone, prednisone, within 1 month before breakthrough infection (dexamethasone was excluded from the list because it is also used to treat severe COVID-19; hydrocortisone and budesonide were excluded due to frequent topical or inhaled use; other glucocorticoids that were also excluded are exclusively topical, inhaled, or injected locally) |
| Leukocyte-inhibitory | Abatacept, azathioprine, baricitinib, belimumab, dimethyl fumarate, fingolimod, leflunomide, methotrexate, mycophenolate, mycophenolic, natalizumab, rapamycin, sirolimus, teriflunomide, tofacitinib, upadacitinib, vedolizumab, within 3 months before breakthrough infection (cyclosporine and tacrolimus were excluded from the list because they are also used topically) |
| Lymphocyte-depleting | Alemtuzumab, obinutuzumab, ocrelizumab, rituximab, within 18 months before breakthrough infection |
| Comorbidities | These comorbidities were derived based the diagnosis and procedure codes described in the CMS Chronic Conditions Warehouse algorithms (<https://www2.ccwdata.org/web/guest/condition-categories>) any time in the 1 year prior to the index date. Conditions studied individually as covariates in this study are noted with an asterisk (*). Several other conditions were included in the groups of comorbidities defined below. |
| *Acquired hypothyroidism |  |
| Acute myocardial infarction |  |
| *Alzheimers disease and related disorders or senile dementia |  |
| *Anemia |  |
| Anxiety disorders |  |
| Asthma |  |
| Atrial fibrillation |  |
| Attention deficit hyperactivity disorder (ADHD) or conduct disorder |  |
| Bipolar disorder |  |
| Cerebral palsy |  |
| *Chronic kidney disease |  |
| *Chronic obstructive pulmonary disease (COPD) and bronchiectasis |  |
| Colorectal cancer |  |
| Depression |  |
| Depressive disorders |  |
| *Diabetes |  |
| Epilepsy |  |
| Endometrial cancer |  |
| Female or male breast cancer |  |
| Heart failure |  |
| Human immunodeficiency virus and/or acquired immunodeficiency syndrome HIV/AIDS |  |
| Hyperlipidemia |  |
| Hypertension |  |
| Ischemic heart disease |  |
| Leukemias and lymphomas |  |
| Liver disease cirrhosis and other liver conditions except viral hepatitis |  |
| Lung cancer |  |
| Mobility impairments |  |
| Multiple sclerosis and transverse myelitis |  |
| Muscular dystrophy |  |
| Peripheral neuropathy |  |
| Peripheral vascular disease |  |
| Post-traumatic stress disorder (PTSD) |  |
| Pressure and chronic ulcers |  |
| Prostate cancer |  |
| Schizophrenia and other psychotic disorders |  |
| Spina bifida or other congenital neurologic |  |
| Spinal cord injury |  |
| Stroke or transient ischemic attack |  |
| Traumatic brain injury |  |
| *Tobacco use |  |
| Viral hepatitis |  |
| Comorbidity groups | Combinations of conditions defined individually using CMS Chronic Conditions Warehouse algorithms (<https://www2.ccwdata.org/web/guest/condition-categories>) any time in the 1 year prior to the date of the viral test. |
| Cancer | Colorectal cancer, endometrial cancer, female or male breast cancer, leukemias and lymphomas, lung cancer, prostate cancer, or cytotoxic chemotherapy (see list of drugs above) |
| Cardiovascular disease | Acute myocardial infarction, atrial fibrillation, heart failure, hyperlipidemia, hypertension, ischemic heart disease, peripheral vascular disease, stroke or transient ischemic attack |
| Impaired function / mobility | Mobility impairments, pressure and chronic ulcers, spinal cord injury |
| Liver disease | Liver disease cirrhosis and other liver conditions except viral hepatitis, viral hepatitis |
| Neurologic disease | Cerebral palsy, epilepsy, multiple sclerosis and transverse myelitis, muscular dystrophy, peripheral neuropathy, spina bifida, traumatic brain injury |
| Psychiatric condition | Anxiety disorders, ADHD conduct disorder and hyperkinetic syndrome, depression, depressive disorders, post-traumatic stress disorder (PTSD), schizophrenia, schizophrenia and other psychotic disorders |
| Vascular disease | A subset of Cardiovascular: acute myocardial infarction, ischemic heart disease, peripheral vascular disease, or stroke or transient ischemic attack |

**sTable 2. Demographic and clinical characteristics of the cohort, stratified by test results for viral infections.** Key demographic and clinical characteristics of subcohorts defined by the results of tests for viral infections. Numbers in parentheses indicate the percentages of patients in the column who had that characteristic. “Multiple tests” indicates any combination of positive tests for SARS-CoV-2, influenza, and RSV during the same week. See sTable 1 for definitions and sTable 3 for additional data for patients with multiple infections.

|  | **All negative** | | **SARS-CoV-2 only** | | **Influenza only** | | **RSV only** | | **Multiple tests** | |
| --- | --- | --- | --- | --- | --- | --- | --- | --- | --- | --- |
|  | **N** | **%** | **N** | **%** | **N** | **%** | **N** | **%** | **N** | **%** |
| **Total, N (%)** | 620434 | (100.0) | 170592 | (100.0) | 30454 | (100.0) | 13207 | (100.0) | 1300 | (100.0) |
| **Age ranges, N (%)** |  |  |  |  |  |  |  |  |  |  |
| **17-<40** | 72717 | (11.7) | 21209 | (12.4) | 5813 | (19.1) | 1409 | (10.7) | 204 | (15.7) |
| **40-<44** | 30723 | (5.0) | 9568 | (5.6) | 2109 | (6.9) | 580 | (4.4) | 76 | (5.8) |
| **45-<50** | 27262 | (4.4) | 8638 | (5.1) | 1756 | (5.8) | 596 | (4.5) | 62 | (4.8) |
| **50-<55** | 48943 | (7.9) | 15085 | (8.8) | 2901 | (9.5) | 1139 | (8.6) | 115 | (8.8) |
| **55-<60** | 52258 | (8.4) | 15161 | (8.9) | 2847 | (9.3) | 1145 | (8.7) | 122 | (9.4) |
| **60-<65** | 72995 | (11.8) | 18460 | (10.8) | 3685 | (12.1) | 1507 | (11.4) | 154 | (11.8) |
| **65-<70** | 74369 | (12.0) | 18418 | (10.8) | 3133 | (10.3) | 1491 | (11.3) | 142 | (10.9) |
| **70-<75** | 112804 | (18.2) | 28224 | (16.5) | 4044 | (13.3) | 2385 | (18.1) | 201 | (15.5) |
| **75-<80** | 68380 | (11.0) | 18528 | (10.9) | 2450 | (8.0) | 1644 | (12.4) | 130 | (10.0) |
| **>=80** | 59909 | (9.7) | 17280 | (10.1) | 1713 | (5.6) | 1309 | (9.9) | 94 | (7.2) |
| **Unknown** | 74 | (0.0) | 21 | (0.0) | 3 | (0.0) | 2 | (0.0) | 0 | 0 |
| **Race, N (%)** |  |  |  |  |  |  |  |  |  |  |
| **American Indian or Alaska Native** | 4972 | (0.8) | 1548 | (0.9) | 272 | (0.9) | 113 | (0.9) | 6 | (0.5) |
| **Asian** | 6382 | (1.0) | 2297 | (1.3) | 346 | (1.1) | 165 | (1.2) | 15 | (1.2) |
| **Black or African American** | 142544 | (23.0) | 37640 | (22.1) | 7587 | (24.9) | 2640 | (20.0) | 240 | (18.5) |
| **More than one race** | 6315 | (1.0) | 1771 | (1.0) | 328 | (1.1) | 133 | (1.0) | 9 | (0.7) |
| **Native Hawaiian or Pacific Islander** | 5334 | (0.9) | 1635 | (1.0) | 307 | (1.0) | 134 | (1.0) | 9 | (0.7) |
| **White** | 408374 | (65.8) | 112365 | (65.9) | 19203 | (63.1) | 9043 | (68.5) | 917 | (70.5) |
| **Unknown** | 46513 | (7.5) | 13336 | (7.8) | 2411 | (7.9) | 979 | (7.4) | 104 | (8.0) |
| **Sex, N (%)** |  |  |  |  |  |  |  |  |  |  |
| **F** | 68863 | (11.1) | 20251 | (11.9) | 4089 | (13.4) | 1706 | (12.9) | 160 | (12.3) |
| **M** | 551497 | (88.9) | 150321 | (88.1) | 26362 | (86.6) | 11500 | (87.1) | 1140 | (87.7) |
| **Unknown** | 74 | (0.0) | 20 | (0.0) | 3 | (0.0) | 1 | (0.0) | 0 | 0 |
| **Ethnicity (Hispanic/Latino), N (%)** |  |  |  |  |  |  |  |  |  |  |
| **Hispanic or Latino** | 54276 | (8.7) | 17997 | (10.5) | 3404 | (11.2) | 1298 | (9.8) | 143 | (11.0) |
| **Not Hispanic or Latino** | 533949 | (86.1) | 143662 | (84.2) | 25488 | (83.7) | 11276 | (85.4) | 1085 | (83.5) |
| **Unknown** | 32209 | (5.2) | 8933 | (5.2) | 1562 | (5.1) | 633 | (4.8) | 72 | (5.5) |
| **Region, N (%)** |  |  |  |  |  |  |  |  |  |  |
| **Continental** | 96046 | (15.5) | 28841 | (16.9) | 5549 | (18.2) | 2060 | (15.6) | 189 | (14.5) |
| **Midwest** | 124046 | (20.0) | 34485 | (20.2) | 6186 | (20.3) | 2745 | (20.8) | 288 | (22.2) |
| **North Atlantic** | 143044 | (23.1) | 35658 | (20.9) | 6402 | (21.0) | 3016 | (22.8) | 270 | (20.8) |
| **Pacific** | 116171 | (18.7) | 34105 | (20.0) | 5503 | (18.1) | 2769 | (21.0) | 307 | (23.6) |
| **Southeast** | 141126 | (22.7) | 37502 | (22.0) | 6814 | (22.4) | 2617 | (19.8) | 246 | (18.9) |
| **Unknown** | 1 | (0.0) | 1 | (0.0) | 0 | 0 | 0 | 0 | 0 | 0 |
| **Vaccine manufacturer (SARS-CoV-2), N (%)** |  |  |  |  |  |  |  |  |  |  |
| **Janssen** | 33350 | (5.4) | 9439 | (5.5) | 1779 | (5.8) | 620 | (4.7) | 77 | (5.9) |
| **Moderna** | 234786 | (37.8) | 62643 | (36.7) | 10780 | (35.4) | 5440 | (41.2) | 489 | (37.6) |
| **Pfizer** | 230777 | (37.2) | 60158 | (35.3) | 10841 | (35.6) | 4895 | (37.1) | 472 | (36.3) |
| **Unknown including unvaccinated** | 121521 | (19.6) | 38352 | (22.5) | 7054 | (23.2) | 2252 | (17.1) | 262 | (20.2) |
| **SARS-CoV-2 infection before vaccination, N (%)** | 25144 | (4.1) | 8055 | (4.7) | 1835 | (6.0) | 766 | (5.8) | 57 | (4.4) |
| **BMI Class, N (%)** |  |  |  |  |  |  |  |  |  |  |
| **Underweight** | 7890 | (1.3) | 1698 | (1.0) | 211 | (0.7) | 128 | (1.0) | 8 | (0.6) |
| **Normal** | 105541 | (17.0) | 26368 | (15.5) | 4116 | (13.5) | 1881 | (14.2) | 178 | (13.7) |
| **Overweight** | 188089 | (30.3) | 52308 | (30.7) | 9072 | (29.8) | 4027 | (30.5) | 388 | (29.8) |
| **Mild Obesity** | 164100 | (26.4) | 46764 | (27.4) | 8651 | (28.4) | 3671 | (27.8) | 375 | (28.8) |
| **Moderate Obesity** | 84817 | (13.7) | 24167 | (14.2) | 4798 | (15.8) | 2050 | (15.5) | 201 | (15.5) |
| **Severe Obesity** | 52222 | (8.4) | 14983 | (8.8) | 2969 | (9.7) | 1264 | (9.6) | 126 | (9.7) |
| **Unknown** | 17775 | (2.9) | 4304 | (2.5) | 637 | (2.1) | 186 | (1.4) | 24 | (1.8) |
| **Immune-suppressive drugs, N (%)** | 97848 | (15.8) | 27231 | (16.0) | 6819 | (22.4) | 4521 | (34.2) | 309 | (23.8) |
| **Cytokine blocking** | 7484 | (1.2) | 2246 | (1.3) | 399 | (1.3) | 235 | (1.8) | 22 | (1.7) |
| **Glucocorticoids** | 85057 | (13.7) | 23099 | (13.5) | 6289 | (20.7) | 4207 | (31.9) | 275 | (21.2) |
| **Leukocyte-inhibitory** | 8458 | (1.4) | 2809 | (1.6) | 353 | (1.2) | 246 | (1.9) | 25 | (1.9) |
| **Lymphocyte-depleting** | 4989 | (0.8) | 2045 | (1.2) | 216 | (0.7) | 181 | (1.4) | 13 | (1.0) |
| **Comorbidities, N (%)** |  |  |  |  |  |  |  |  |  |  |
| **Comorbidity groups and components** |  |  |  |  |  |  |  |  |  |  |
| **Cancer** | 72634 | (11.7) | 18754 | (11.0) | 2612 | (8.6) | 1579 | (12.0) | 144 | (11.1) |
| **Breast cancer** | 2836 | (0.5) | 687 | (0.4) | 113 | (0.4) | 74 | (0.6) | 9 | (0.7) |
| **Colorectal cancer** | 7870 | (1.3) | 1941 | (1.1) | 261 | (0.9) | 155 | (1.2) | 12 | (0.9) |
| **Endometrial cancer** | 175 | (0.0) | 46 | (0.0) | 9 | (0.0) | 6 | (0.0) | 0 | 0 |
| **Leukemia / lymphoma** | 10409 | (1.7) | 3291 | (1.9) | 433 | (1.4) | 274 | (2.1) | 22 | (1.7) |
| **Lung cancer** | 12271 | (2.0) | 2618 | (1.5) | 398 | (1.3) | 239 | (1.8) | 25 | (1.9) |
| **Prostate cancer** | 40926 | (6.6) | 10755 | (6.3) | 1465 | (4.8) | 864 | (6.5) | 79 | (6.1) |
| **Chemotherapy** | 8001 | (1.3) | 1803 | (1.1) | 208 | (0.7) | 156 | (1.2) | 7 | (0.5) |
| **Cardiovascular disease** | 446759 | (72.0) | 122539 | (71.8) | 20050 | (65.8) | 9936 | (75.2) | 904 | (69.5) |
| **Atrial fibrillation** | 63761 | (10.3) | 17344 | (10.2) | 2328 | (7.6) | 1577 | (11.9) | 125 | (9.6) |
| **Heart failure** | 62705 | (10.1) | 17454 | (10.2) | 2412 | (7.9) | 1588 | (12.0) | 122 | (9.4) |
| **Hyperlipidemia** | 317148 | (51.1) | 89708 | (52.6) | 14609 | (48.0) | 7359 | (55.7) | 687 | (52.8) |
| **Hypertension** | 359869 | (58.0) | 97483 | (57.1) | 15611 | (51.3) | 7976 | (60.4) | 681 | (52.4) |
| **Vascular disease** | 158008 | (25.5) | 42628 | (25.0) | 5993 | (19.7) | 3441 | (26.1) | 284 | (21.8) |
| **Acute myocardial infarction** | 10496 | (1.7) | 3770 | (2.2) | 488 | (1.6) | 306 | (2.3) | 16 | (1.2) |
| **Ischemic heart disease** | 119223 | (19.2) | 32722 | (19.2) | 4634 | (15.2) | 2740 | (20.7) | 230 | (17.7) |
| **Peripheral vascular disease** | 45176 | (7.3) | 11770 | (6.9) | 1564 | (5.1) | 908 | (6.9) | 81 | (6.2) |
| **Stroke / TIA** | 29832 | (4.8) | 8484 | (5.0) | 1047 | (3.4) | 653 | (4.9) | 52 | (4.0) |
| **Impaired function / mobility** | 33188 | (5.3) | 9095 | (5.3) | 976 | (3.2) | 605 | (4.6) | 54 | (4.2) |
| **Mobility impairments** | 13474 | (2.2) | 3657 | (2.1) | 372 | (1.2) | 234 | (1.8) | 26 | (2.0) |
| **Pressure / chronic ulcers** | 22033 | (3.6) | 5975 | (3.5) | 629 | (2.1) | 403 | (3.1) | 29 | (2.2) |
| **Spinal cord injury** | 2574 | (0.4) | 697 | (0.4) | 82 | (0.3) | 50 | (0.4) | 5 | (0.4) |
| **Liver disease** | 56054 | (9.0) | 15137 | (8.9) | 2517 | (8.3) | 1147 | (8.7) | 118 | (9.1) |
| **Liver cirrhosis and other non-viral** | 45614 | (7.4) | 12587 | (7.4) | 2062 | (6.8) | 964 | (7.3) | 94 | (7.2) |
| **Viral hepatitis** | 16052 | (2.6) | 4044 | (2.4) | 647 | (2.1) | 274 | (2.1) | 34 | (2.6) |
| **Neurologic disease** | 110882 | (17.9) | 31508 | (18.5) | 4608 | (15.1) | 2550 | (19.3) | 216 | (16.6) |
| **Cerebral palsy** | 51 | (0.0) | 12 | (0.0) | 0 | 0 | 2 | (0.0) | 0 | 0 |
| **Epilepsy** | 13628 | (2.2) | 3676 | (2.2) | 472 | (1.5) | 238 | (1.8) | 14 | (1.1) |
| **Multiple sclerosis / transverse myelitis** | 2790 | (0.4) | 928 | (0.5) | 111 | (0.4) | 47 | (0.4) | 3 | (0.2) |
| **Muscular dystrophy** | 207 | (0.0) | 69 | (0.0) | 10 | (0.0) | 4 | (0.0) | 0 | 0 |
| **Peripheral neuropathy** | 95433 | (15.4) | 27258 | (16.0) | 3984 | (13.1) | 2290 | (17.3) | 200 | (15.4) |
| **Spina bifida / congenital neurologic** | 482 | (0.1) | 168 | (0.1) | 31 | (0.1) | 8 | (0.1) | 3 | (0.2) |
| **Traumatic brain injury** | 3476 | (0.6) | 1040 | (0.6) | 163 | (0.5) | 65 | (0.5) | 4 | (0.3) |
| **Psychiatric disorder** | 293371 | (47.3) | 80500 | (47.2) | 14853 | (48.8) | 6271 | (47.5) | 600 | (46.2) |
| **Anxiety disorders** | 215795 | (34.8) | 60251 | (35.3) | 11410 | (37.5) | 4746 | (35.9) | 452 | (34.8) |
| **ADHD / Conduct disorder** | 14512 | (2.3) | 4330 | (2.5) | 903 | (3.0) | 319 | (2.4) | 40 | (3.1) |
| **Bipolar disorder** | 57215 | (9.2) | 15521 | (9.1) | 2748 | (9.0) | 1174 | (8.9) | 127 | (9.8) |
| **Depression** | 183346 | (29.6) | 50626 | (29.7) | 9133 | (30.0) | 3862 | (29.2) | 359 | (27.6) |
| **Depressive disorders** | 165402 | (26.7) | 45699 | (26.8) | 8186 | (26.9) | 3434 | (26.0) | 318 | (24.5) |
| **PTSD** | 142626 | (23.0) | 39244 | (23.0) | 7440 | (24.4) | 3033 | (23.0) | 287 | (22.1) |
| **Schizophrenia / psychosis** | 27498 | (4.4) | 6865 | (4.0) | 1033 | (3.4) | 455 | (3.4) | 34 | (2.6) |
| **Comorbidities, other ungrouped** |  |  |  |  |  |  |  |  |  |  |
| **Alzheimer's / dementia** | 40523 | (6.5) | 12717 | (7.5) | 1198 | (3.9) | 874 | (6.6) | 76 | (5.8) |
| **Anemia** | 99474 | (16.0) | 28520 | (16.7) | 3805 | (12.5) | 2198 | (16.6) | 176 | (13.5) |
| **Asthma** | 35164 | (5.7) | 10830 | (6.3) | 2534 | (8.3) | 1167 | (8.8) | 107 | (8.2) |
| **Chronic kidney disease** | 119083 | (19.2) | 35232 | (20.7) | 4867 | (16.0) | 2822 | (21.4) | 218 | (16.8) |
| **COPD / bronchiectasis** | 102000 | (16.4) | 25719 | (15.1) | 4719 | (15.5) | 2491 | (18.9) | 211 | (16.2) |
| **Diabetes** | 196016 | (31.6) | 53990 | (31.6) | 8269 | (27.2) | 4297 | (32.5) | 371 | (28.5) |
| **HIV / AIDS** | 6174 | (1.0) | 1443 | (0.8) | 373 | (1.2) | 177 | (1.3) | 14 | (1.1) |
| **Hypothyroidism** | 53580 | (8.6) | 15438 | (9.0) | 2371 | (7.8) | 1282 | (9.7) | 109 | (8.4) |
| **Tobacco use** | 121542 | (19.6) | 28094 | (16.5) | 5761 | (18.9) | 2119 | (16.0) | 220 | (16.9) |

**sTable 3. Demographic and clinical characteristics of patients with multiple viral infections.** Key demographic and clinical characteristics of patients with positive tests for more than one viral infection. Numbers in parentheses indicate the percentages of patients in the column who had that characteristic. See sTable 1 for definitions.

|  | **Multiple Tests (any)** | | **Influenza + RSV** | | **SARS-CoV-2 + RSV** | | **SARS-CoV-2 + Influenza** | |
| --- | --- | --- | --- | --- | --- | --- | --- | --- |
|  | **N** | **%** | **N** | **%** | **N** | **%** | **N** | **%** |
| **Total, N (%)** | 1300 | (100.0) | 131 | (100.0) | 425 | (100.0) | 742 | (100.0) |
| **Age ranges, N (%)** |  |  |  |  |  |  |  |  |
| **17-<40** | 204 | (15.7) | 24 | (18.3) | 55 | (12.9) | 124 | (16.7) |
| **40-<44** | 76 | (5.8) | 10 | (7.6) | 20 | (4.7) | 46 | (6.2) |
| **45-<50** | 62 | (4.8) | 9 | (6.9) | 16 | (3.8) | 36 | (4.9) |
| **50-<55** | 115 | (8.8) | 11 | (8.4) | 34 | (8.0) | 70 | (9.4) |
| **55-<60** | 122 | (9.4) | 9 | (6.9) | 47 | (11.1) | 66 | (8.9) |
| **60-<65** | 154 | (11.8) | 13 | (9.9) | 49 | (11.5) | 92 | (12.4) |
| **65-<70** | 142 | (10.9) | 16 | (12.2) | 45 | (10.6) | 81 | (10.9) |
| **70-<75** | 201 | (15.5) | 20 | (15.3) | 70 | (16.5) | 111 | (15.0) |
| **75-<80** | 130 | (10.0) | 9 | (6.9) | 48 | (11.3) | 73 | (9.8) |
| **>=80** | 94 | (7.2) | 10 | (7.6) | 41 | (9.6) | 43 | (5.8) |
| **Unknown** | 0 | 0 | 0 | 0 | 0 | 0 | 0 | 0 |
| **Race, N (%)** |  |  |  |  |  |  |  |  |
| **American Indian or Alaska Native** | 6 | (0.5) | 0 | 0 | 3 | (0.7) | 3 | (0.4) |
| **Asian** | 15 | (1.2) | 4 | (3.1) | 5 | (1.2) | 6 | (0.8) |
| **Black or African American** | 240 | (18.5) | 27 | (20.6) | 62 | (14.6) | 151 | (20.4) |
| **More than one race** | 9 | (0.7) | 1 | (0.8) | 1 | (0.2) | 7 | (0.9) |
| **Native Hawaiian or Pacific Islander** | 9 | (0.7) | 2 | (1.5) | 4 | (0.9) | 3 | (0.4) |
| **White** | 917 | (70.5) | 88 | (67.2) | 316 | (74.4) | 511 | (68.9) |
| **Unknown** | 104 | (8.0) | 9 | (6.9) | 34 | (8.0) | 61 | (8.2) |
| **Sex, N (%)** |  |  |  |  |  |  |  |  |
| **F** | 160 | (12.3) | 15 | (11.5) | 48 | (11.3) | 96 | (12.9) |
| **M** | 1140 | (87.7) | 116 | (88.5) | 377 | (88.7) | 646 | (87.1) |
| **Unknown** | 0 | 0 | 0 | 0 | 0 | 0 | 0 | 0 |
| **Ethnicity (Hispanic/Latino), N (%)** |  |  |  |  |  |  |  |  |
| **Hispanic or Latino** | 143 | (11.0) | 11 | (8.4) | 46 | (10.8) | 86 | (11.6) |
| **Not Hispanic or Latino** | 1085 | (83.5) | 116 | (88.5) | 351 | (82.6) | 616 | (83.0) |
| **Unknown** | 72 | (5.5) | 4 | (3.1) | 28 | (6.6) | 40 | (5.4) |
| **Region, N (%)** |  |  |  |  |  |  |  |  |
| **Continental** | 189 | (14.5) | 25 | (19.1) | 66 | (15.5) | 98 | (13.2) |
| **Midwest** | 288 | (22.2) | 31 | (23.7) | 82 | (19.3) | 174 | (23.5) |
| **North Atlantic** | 270 | (20.8) | 27 | (20.6) | 76 | (17.9) | 166 | (22.4) |
| **Pacific** | 307 | (23.6) | 27 | (20.6) | 142 | (33.4) | 138 | (18.6) |
| **Southeast** | 246 | (18.9) | 21 | (16.0) | 59 | (13.9) | 166 | (22.4) |
| **Unknown** | 0 | 0 | 0 | 0 | 0 | 0 | 0 | 0 |
| **Vaccine manufacturer (SARS-CoV-2), N (%)** |  |  |  |  |  |  |  |  |
| **Janssen** | 77 | (5.9) | 5 | (3.8) | 26 | (6.1) | 46 | (6.2) |
| **Moderna** | 489 | (37.6) | 51 | (38.9) | 156 | (36.7) | 281 | (37.9) |
| **Pfizer** | 472 | (36.3) | 46 | (35.1) | 169 | (39.8) | 256 | (34.5) |
| **Unknown including unvaccinated** | 262 | (20.2) | 29 | (22.1) | 74 | (17.4) | 159 | (21.4) |
| **SARS-CoV-2 infection before vaccination, N (%)** | 57 | (4.4) | 3 | (2.3) | 17 | (4.0) | 37 | (5.0) |
| **BMI Class, N (%)** |  |  |  |  |  |  |  |  |
| **Underweight** | 8 | (0.6) | 0 | 0 | 1 | (0.2) | 7 | (0.9) |
| **Normal** | 178 | (13.7) | 19 | (14.5) | 67 | (15.8) | 92 | (12.4) |
| **Overweight** | 388 | (29.8) | 34 | (26.0) | 128 | (30.1) | 225 | (30.3) |
| **Mild Obesity** | 375 | (28.8) | 36 | (27.5) | 124 | (29.2) | 214 | (28.8) |
| **Moderate Obesity** | 201 | (15.5) | 24 | (18.3) | 58 | (13.6) | 119 | (16.0) |
| **Severe Obesity** | 126 | (9.7) | 16 | (12.2) | 37 | (8.7) | 73 | (9.8) |
| **Unknown** | 24 | (1.8) | 2 | (1.5) | 10 | (2.4) | 12 | (1.6) |
| **Immune-suppressive drugs, N (%)** | 309 | (23.8) | 37 | (28.2) | 93 | (21.9) | 178 | (24.0) |
| **Cytokine blocking** | 22 | (1.7) | 1 | (0.8) | 10 | (2.4) | 11 | (1.5) |
| **Glucocorticoids** | 275 | (21.2) | 35 | (26.7) | 79 | (18.6) | 160 | (21.6) |
| **Leukocyte-inhibitory** | 25 | (1.9) | 1 | (0.8) | 9 | (2.1) | 15 | (2.0) |
| **Lymphocyte-depleting** | 13 | (1.0) | 2 | (1.5) | 4 | (0.9) | 7 | (0.9) |
| **Comorbidities, N (%)** |  |  |  |  |  |  |  |  |
| **Comorbidity groups and components** |  |  |  |  |  |  |  |  |
| **Cancer** | 144 | (11.1) | 13 | (9.9) | 51 | (12.0) | 80 | (10.8) |
| **Breast cancer** | 9 | (0.7) | 0 | 0 | 4 | (0.9) | 5 | (0.7) |
| **Colorectal cancer** | 12 | (0.9) | 0 | 0 | 6 | (1.4) | 6 | (0.8) |
| **Endometrial cancer** | 0 | 0 | 0 | 0 | 0 | 0 | 0 | 0 |
| **Leukemia / lymphoma** | 22 | (1.7) | 5 | (3.8) | 9 | (2.1) | 8 | (1.1) |
| **Lung cancer** | 25 | (1.9) | 1 | (0.8) | 5 | (1.2) | 19 | (2.6) |
| **Prostate cancer** | 79 | (6.1) | 7 | (5.3) | 29 | (6.8) | 43 | (5.8) |
| **Chemotherapy** | 7 | (0.5) | 2 | (1.5) | 2 | (0.5) | 3 | (0.4) |
| **Cardiovascular disease** | 904 | (69.5) | 85 | (64.9) | 312 | (73.4) | 506 | (68.2) |
| **Atrial fibrillation** | 125 | (9.6) | 14 | (10.7) | 45 | (10.6) | 66 | (8.9) |
| **Heart failure** | 122 | (9.4) | 12 | (9.2) | 48 | (11.3) | 62 | (8.4) |
| **Hyperlipidemia** | 687 | (52.8) | 67 | (51.1) | 239 | (56.2) | 380 | (51.2) |
| **Hypertension** | 681 | (52.4) | 63 | (48.1) | 237 | (55.8) | 381 | (51.3) |
| **Vascular disease** | 284 | (21.8) | 24 | (18.3) | 101 | (23.8) | 159 | (21.4) |
| **Acute myocardial infarction** | 16 | (1.2) | 1 | (0.8) | 7 | (1.6) | 8 | (1.1) |
| **Ischemic heart disease** | 230 | (17.7) | 21 | (16.0) | 72 | (16.9) | 137 | (18.5) |
| **Peripheral vascular disease** | 81 | (6.2) | 5 | (3.8) | 34 | (8.0) | 42 | (5.7) |
| **Stroke / TIA** | 52 | (4.0) | 5 | (3.8) | 20 | (4.7) | 27 | (3.6) |
| **Impaired function / mobility** | 54 | (4.2) | 5 | (3.8) | 25 | (5.9) | 24 | (3.2) |
| **Mobility impairments** | 26 | (2.0) | 2 | (1.5) | 11 | (2.6) | 13 | (1.8) |
| **Pressure / chronic ulcers** | 29 | (2.2) | 3 | (2.3) | 15 | (3.5) | 11 | (1.5) |
| **Spinal cord injury** | 5 | (0.4) | 1 | (0.8) | 4 | (0.9) | 0 | 0 |
| **Liver disease** | 118 | (9.1) | 17 | (13.0) | 30 | (7.1) | 70 | (9.4) |
| **Liver cirrhosis and other non-viral** | 94 | (7.2) | 14 | (10.7) | 21 | (4.9) | 58 | (7.8) |
| **Viral hepatitis** | 34 | (2.6) | 5 | (3.8) | 12 | (2.8) | 17 | (2.3) |
| **Neurologic disease** | 216 | (16.6) | 23 | (17.6) | 75 | (17.6) | 118 | (15.9) |
| **Cerebral palsy** | 0 | 0 | 0 | 0 | 0 | 0 | 0 | 0 |
| **Epilepsy** | 14 | (1.1) | 0 | 0 | 5 | (1.2) | 9 | (1.2) |
| **Multiple sclerosis / transverse myelitis** | 3 | (0.2) | 0 | 0 | 2 | (0.5) | 1 | (0.1) |
| **Muscular dystrophy** | 0 | 0 | 0 | 0 | 0 | 0 | 0 | 0 |
| **Peripheral neuropathy** | 200 | (15.4) | 22 | (16.8) | 68 | (16.0) | 110 | (14.8) |
| **Spina bifida / congenital neurologic** | 3 | (0.2) | 0 | 0 | 2 | (0.5) | 1 | (0.1) |
| **Traumatic brain injury** | 4 | (0.3) | 1 | (0.8) | 0 | 0 | 3 | (0.4) |
| **Psychiatric disorder** | 600 | (46.2) | 55 | (42.0) | 205 | (48.2) | 339 | (45.7) |
| **Anxiety disorders** | 452 | (34.8) | 43 | (32.8) | 149 | (35.1) | 259 | (34.9) |
| **ADHD / Conduct disorder** | 40 | (3.1) | 4 | (3.1) | 13 | (3.1) | 23 | (3.1) |
| **Bipolar disorder** | 127 | (9.8) | 9 | (6.9) | 41 | (9.6) | 77 | (10.4) |
| **Depression** | 359 | (27.6) | 26 | (19.8) | 127 | (29.9) | 205 | (27.6) |
| **Depressive disorders** | 318 | (24.5) | 26 | (19.8) | 115 | (27.1) | 176 | (23.7) |
| **PTSD** | 287 | (22.1) | 25 | (19.1) | 104 | (24.5) | 157 | (21.2) |
| **Schizophrenia / psychosis** | 34 | (2.6) | 3 | (2.3) | 10 | (2.4) | 21 | (2.8) |
| **Comorbidities, other ungrouped** |  |  |  |  |  |  |  |  |
| **Alzheimer's / dementia** | 76 | (5.8) | 5 | (3.8) | 29 | (6.8) | 42 | (5.7) |
| **Anemia** | 176 | (13.5) | 17 | (13.0) | 66 | (15.5) | 93 | (12.5) |
| **Asthma** | 107 | (8.2) | 12 | (9.2) | 31 | (7.3) | 63 | (8.5) |
| **Chronic kidney disease** | 218 | (16.8) | 22 | (16.8) | 77 | (18.1) | 119 | (16.0) |
| **COPD / bronchiectasis** | 211 | (16.2) | 27 | (20.6) | 64 | (15.1) | 119 | (16.0) |
| **Diabetes** | 371 | (28.5) | 34 | (26.0) | 126 | (29.6) | 211 | (28.4) |
| **HIV / AIDS** | 14 | (1.1) | 0 | 0 | 3 | (0.7) | 11 | (1.5) |
| **Hypothyroidism** | 109 | (8.4) | 8 | (6.1) | 35 | (8.2) | 65 | (8.8) |
| **Tobacco use** | 220 | (16.9) | 16 | (12.2) | 65 | (15.3) | 138 | (18.6) |

**sTable 4.** **Hypoxemia among patients with different test results for viral infection.** Numbers in parentheses indicate the percentages of patients with that variable/characteristic who experienced hypoxemia within 14 days of the index date.

| **Virus test result** | **All negative** | | **SARS-CoV-2 only** | | **Influenza only** | | **RSV only** | | **Multiple tests** | |
| --- | --- | --- | --- | --- | --- | --- | --- | --- | --- | --- |
| **Hypoxemia** | **No** | **Yes** | **No** | **Yes** | **No** | **Yes** | **No** | **Yes** | **No** | **Yes** |
| **Total, N (%)** | 565299 | 55135 (8.9) | 156924 | 13668 (8.0) | 28104 | 2350 (7.7) | 11985 | 1222 (9.3) | 1187 | 113 (8.7) |
| **Age ranges** |  |  |  |  |  |  |  |  |  |  |
| **17-<40** | 71653 | 1064 (1.5) | 21018 | 191 (0.9) | 5777 | 36 (0.6) | 1393 | 16 (1.1) | 203 | 1 (0.5) |
| **40-<45** | 30131 | 592 (1.9) | 9479 | 89 (0.9) | 2088 | 21 (1.0) | 575 | 5 (0.9) | 76 | 0 |
| **45-<50** | 26584 | 678 (2.5) | 8502 | 136 (1.6) | 1726 | 30 (1.7) | 582 | 14 (2.3) | 61 | 1 (1.6) |
| **50-<55** | 47151 | 1792 (3.7) | 14723 | 362 (2.4) | 2817 | 84 (2.9) | 1106 | 33 (2.9) | 111 | 4 (3.5) |
| **55-<60** | 49418 | 2840 (5.4) | 14585 | 576 (3.8) | 2730 | 117 (4.1) | 1091 | 54 (4.7) | 118 | 4 (3.3) |
| **60-<65** | 67179 | 5816 (8.0) | 17335 | 1125 (6.1) | 3371 | 314 (8.5) | 1374 | 133 (8.8) | 143 | 11 (7.1) |
| **65-<70** | 66479 | 7890 (10.6) | 16635 | 1783 (9.7) | 2765 | 368 (11.7) | 1319 | 172 (11.5) | 128 | 14 (9.9) |
| **70-<75** | 98674 | 14130 (12.5) | 24750 | 3474 (12.3) | 3473 | 571 (14.1) | 2070 | 315 (13.2) | 163 | 38 (18.9) |
| **75-<80** | 58730 | 9650 (14.1) | 15937 | 2591 (14.0) | 1995 | 455 (18.6) | 1408 | 236 (14.4) | 112 | 18 (13.8) |
| **>=80** | 49226 | 10683 (17.8) | 13939 | 3341 (19.3) | 1359 | 354 (20.7) | 1065 | 244 (18.6) | 72 | 22 (23.4) |
| **Unknown** | 74 | 0 | 21 | 0 | 3 | 0 | 2 | 0 | 0 | 0 |
| **Race** |  |  |  |  |  |  |  |  |  |  |
| **American Indian or Alaska Native** | 4566 | 406 (8.2) | 1443 | 105 (6.8) | 261 | 11 (4.0) | 100 | 13 (11.5) | 6 | 0 |
| **Asian** | 6139 | 243 (3.8) | 2230 | 67 (2.9) | 332 | 14 (4.0) | 162 | 3 (1.8) | 15 | 0 |
| **Black or African American** | 133077 | 9467 (6.6) | 35518 | 2122 (5.6) | 7179 | 408 (5.4) | 2456 | 184 (7.0) | 229 | 11 (4.6) |
| **More than one race** | 5881 | 434 (6.9) | 1677 | 94 (5.3) | 311 | 17 (5.2) | 126 | 7 (5.3) | 7 | 2 (22.2) |
| **Native Hawaiian or Pacific Islander** | 4943 | 391 (7.3) | 1539 | 96 (5.9) | 292 | 15 (4.9) | 124 | 10 (7.5) | 8 | 1 (11.1) |
| **White** | 367720 | 40654 (10.0) | 102035 | 10330 (9.2) | 17451 | 1752 (9.1) | 8122 | 921 (10.2) | 823 | 94 (10.3) |
| **Unknown** | 42973 | 3540 (7.6) | 12482 | 854 (6.4) | 2278 | 133 (5.5) | 895 | 84 (8.6) | 99 | 5 (4.8) |
| **Sex** |  |  |  |  |  |  |  |  |  |  |
| **F** | 65888 | 2975 (4.3) | 19694 | 557 (2.8) | 3924 | 165 (4.0) | 1623 | 83 (4.9) | 153 | 7 (4.4) |
| **M** | 499337 | 52160 (9.5) | 137210 | 13111 (8.7) | 24177 | 2185 (8.3) | 10361 | 1139 (9.9) | 1034 | 106 (9.3) |
| **Unknown** | 74 | 0 | 20 | 0 | 3 | 0 | 1 | 0 | 0 | 0 |
| **Ethnicity (Hispanic/Latino)** |  |  |  |  |  |  |  |  |  |  |
| **Hispanic or Latino** | 50908 | 3368 (6.2) | 16775 | 1222 (6.8) | 3259 | 145 (4.3) | 1206 | 92 (7.1) | 133 | 10 (7.0) |
| **Not Hispanic or Latino** | 484735 | 49214 (9.2) | 131793 | 11869 (8.3) | 23385 | 2103 (8.3) | 10200 | 1076 (9.5) | 988 | 97 (8.9) |
| **Unknown** | 29656 | 2553 (7.9) | 8356 | 577 (6.5) | 1460 | 102 (6.5) | 579 | 54 (8.5) | 66 | 6 (8.3) |
| **Region** |  |  |  |  |  |  |  |  |  |  |
| **Continental** | 85256 | 10790 (11.2) | 26340 | 2501 (8.7) | 5060 | 489 (8.8) | 1855 | 205 (10.0) | 172 | 17 (9.0) |
| **Midwest** | 113924 | 10122 (8.2) | 32234 | 2251 (6.5) | 5752 | 434 (7.0) | 2504 | 241 (8.8) | 259 | 29 (10.1) |
| **North Atlantic** | 132478 | 10566 (7.4) | 33081 | 2577 (7.2) | 5988 | 414 (6.5) | 2783 | 233 (7.7) | 252 | 18 (6.7) |
| **Pacific** | 103695 | 12476 (10.7) | 31050 | 3055 (9.0) | 4952 | 551 (10.0) | 2459 | 310 (11.2) | 280 | 27 (8.8) |
| **Southeast** | 129945 | 11181 (7.9) | 34218 | 3284 (8.8) | 6352 | 462 (6.8) | 2384 | 233 (8.9) | 224 | 22 (8.9) |
| **Unknown** | 1 | 0 | 1 | 0 | 0 | 0 | 0 | 0 | 0 | 0 |
| **Vaccine manufacturer (SARS-CoV-2)** |  |  |  |  |  |  |  |  |  |  |
| **Janssen** | 30651 | 2699 (8.1) | 8794 | 645 (6.8) | 1648 | 131 (7.4) | 582 | 38 (6.1) | 75 | 2 (2.6) |
| **Moderna** | 212451 | 22335 (9.5) | 57677 | 4966 (7.9) | 9823 | 957 (8.9) | 4925 | 515 (9.5) | 431 | 58 (11.9) |
| **Pfizer** | 209418 | 21359 (9.3) | 55474 | 4684 (7.8) | 9934 | 907 (8.4) | 4392 | 503 (10.3) | 440 | 32 (6.8) |
| **Unknown including unvaccinated** | 112779 | 8742 (7.2) | 34979 | 3373 (8.8) | 6699 | 355 (5.0) | 2086 | 166 (7.4) | 241 | 21 (8.0) |
| **SARS-CoV-2 infection before vaccination** | 22937 | 2207 (8.8) | 7418 | 637 (7.9) | 1709 | 126 (6.9) | 689 | 77 (10.1) | 53 | 4 (7.0) |
| **BMI class** |  |  |  |  |  |  |  |  |  |  |
| **Underweight** | 5989 | 1901 (24.1) | 1228 | 470 (27.7) | 152 | 59 (28.0) | 80 | 48 (37.5) | 3 | 5 (62.5) |
| **Normal** | 92711 | 12830 (12.2) | 23097 | 3271 (12.4) | 3569 | 547 (13.3) | 1607 | 274 (14.6) | 154 | 24 (13.5) |
| **Overweight** | 173137 | 14952 (7.9) | 48376 | 3932 (7.5) | 8412 | 660 (7.3) | 3683 | 344 (8.5) | 355 | 33 (8.5) |
| **Mild obesity** | 152266 | 11834 (7.2) | 43854 | 2910 (6.2) | 8115 | 536 (6.2) | 3412 | 259 (7.1) | 349 | 26 (6.9) |
| **Moderate obesity** | 78045 | 6772 (8.0) | 22556 | 1611 (6.7) | 4509 | 289 (6.0) | 1903 | 147 (7.2) | 186 | 15 (7.5) |
| **Severe obesity** | 46608 | 5614 (10.8) | 13715 | 1268 (8.5) | 2732 | 237 (8.0) | 1123 | 141 (11.2) | 116 | 10 (7.9) |
| **Unknown** | 16543 | 1232 (6.9) | 4098 | 206 (4.8) | 615 | 22 (3.5) | 177 | 9 (4.8) | 24 | 0 |
| **Immune-suppressive drugs** | 82073 | 15775 (16.1) | 23028 | 4203 (15.4) | 5623 | 1196 (17.5) | 3785 | 736 (16.3) | 259 | 50 (16.2) |
| **Cytokine-blocking** | 6787 | 697 (9.3) | 2001 | 245 (10.9) | 363 | 36 (9.0) | 214 | 21 (8.9) | 21 | 1 (4.5) |
| **Glucocorticoids** | 70493 | 14564 (17.1) | 19394 | 3705 (16.0) | 5137 | 1152 (18.3) | 3491 | 716 (17.0) | 226 | 49 (17.8) |
| **Leukocyte-inhibitory** | 7451 | 1007 (11.9) | 2375 | 434 (15.5) | 309 | 44 (12.5) | 213 | 33 (13.4) | 23 | 2 (8.0) |
| **Lymphocyte-depleting** | 4300 | 689 (13.8) | 1638 | 407 (19.9) | 179 | 37 (17.1) | 155 | 26 (14.4) | 12 | 1 (7.7) |
| **Comorbidities** |  |  |  |  |  |  |  |  |  |  |
| **Comorbidity groups and components** |  |  |  |  |  |  |  |  |  |  |
| **Cancer** | 62595 | 10039 (13.8) | 16064 | 2690 (14.3) | 2231 | 381 (14.6) | 1331 | 248 (15.7) | 119 | 25 (17.4) |
| **Breast cancer** | 2566 | 270 (9.5) | 633 | 54 (7.9) | 108 | 5 (4.4) | 67 | 7 (9.5) | 8 | 1 (11.1) |
| **Colorectal cancer** | 6768 | 1102 (14.0) | 1647 | 294 (15.1) | 222 | 39 (14.9) | 135 | 20 (12.9) | 11 | 1 (8.3) |
| **Endometrial cancer** | 155 | 20 (11.4) | 44 | 2 (4.3) | 8 | 1 (11.1) | 6 | 0 | 0 | 0 |
| **Leukemias / Lymphomas** | 8917 | 1492 (14.3) | 2733 | 558 (17.0) | 380 | 53 (12.2) | 240 | 34 (12.4) | 20 | 2 (9.1) |
| **Lung cancer** | 9131 | 3140 (25.6) | 1918 | 700 (26.7) | 286 | 112 (28.1) | 169 | 70 (29.3) | 16 | 9 (36.0) |
| **Prostate cancer** | 36384 | 4542 (11.1) | 9509 | 1246 (11.6) | 1273 | 192 (13.1) | 743 | 121 (14.0 | 65 | 14 (17.7) |
| **Chemotherapy** | 6549 | 1452 (18.1) | 1432 | 371 (20.6) | 173 | 35 (16.8) | 119 | 37 (23.7) | 5 | 2 (28.6) |
| **Cardiovascular disease** | 400510 | 46249 (10.4) | 110637 | 11902 (9.7) | 18037 | 2013 (10.0) | 8837 | 1099 (11.1) | 802 | 102 (11.3) |
| **Atrial fibrillation** | 52509 | 11252 (17.6) | 14136 | 3208 (18.5) | 1864 | 464 (19.9) | 1236 | 341 (21.6) | 95 | 30 (24.0) |
| **Heart failure** | 48906 | 13799 (22.0) | 13415 | 4039 (23.1) | 1793 | 619 (25.7) | 1185 | 403 (25.4) | 93 | 29 (23.8) |
| **Hyperlipidemia** | 285375 | 31773 (10.0) | 81144 | 8564 (9.5) | 13181 | 1428 (9.8) | 6550 | 809 (11.0) | 615 | 72 (10.5) |
| **Hypertension** | 320152 | 39717 (11.0) | 87098 | 10385 (10.7) | 13868 | 1743 (11.2) | 7026 | 950 (11.9) | 593 | 88 (12.9) |
| **Vascular disease** | 135155 | 22853 (14.5) | 36222 | 6406 (15.0) | 4977 | 1016 (17.0) | 2871 | 570 (16.6) | 236 | 48 (16.9) |
| **Acute myocardial infarction** | 8394 | 2102 (20.0) | 2955 | 815 (21.6) | 362 | 126 (25.8) | 236 | 70 (22.9) | 10 | 6 (37.5) |
| **Ischemic heart disease** | 101950 | 17273 (14.5) | 27840 | 4882 (14.9) | 3842 | 792 (17.1) | 2293 | 447 (16.3) | 188 | 42 (18.3) |
| **Peripheral vascular disease** | 37217 | 7959 (17.6) | 9452 | 2318 (19.7) | 1229 | 335 (21.4) | 709 | 199 (21.9) | 65 | 16 (19.8) |
| **Stroke / TIA** | 25591 | 4241 (14.2) | 7092 | 1392 (16.4) | 877 | 170 (16.2) | 550 | 103 (15.8) | 41 | 11 (21.2) |
| **Impaired function / mobility** | 26798 | 6390 (19.3) | 6982 | 2113 (23.2) | 736 | 240 (24.6) | 461 | 144 (23.8) | 40 | 14 (25.9) |
| **Mobility impairments** | 11240 | 2234 (16.6) | 2921 | 736 (20.1) | 296 | 76 (20.4) | 188 | 46 (19.7) | 22 | 4 (15.4) |
| **Pressure / chronic ulcers** | 17318 | 4715 (21.4) | 4421 | 1554 (26.0) | 451 | 178 (28.3) | 292 | 111 (27.5) | 18 | 11 (37.9) |
| **Spinal cord injury** | 2169 | 405 (15.7) | 556 | 141 (20.2) | 68 | 14 (17.1) | 39 | 11 (22.0) | 5 | 0 |
| **Liver disease** | 50143 | 5911 (10.5) | 13496 | 1641 (10.8) | 2244 | 273 (10.8) | 994 | 153 (13.3) | 104 | 14 (11.9) |
| **Liver cirrhosis, other non-viral** | 40841 | 4773 (10.5) | 11235 | 1352 (10.7) | 1845 | 217 (10.5) | 844 | 120 (12.4) | 82 | 12 (12.8) |
| **Viral hepatitis** | 14198 | 1854 (11.5) | 3576 | 468 (11.6) | 563 | 84 (13.0) | 221 | 53 (19.3) | 29 | 5 (14.7) |
| **Neurologic disease** | 97577 | 13305 (12.0) | 27572 | 3936 (12.5) | 4019 | 589 (12.8) | 2179 | 371 (14.5) | 187 | 29 (13.4) |
| **Cerebral palsy** | 43 | 8 (15.7) | 9 | 3 (25.0) | 0 | 0 | 2 | 0 | 0 | 0 |
| **Epilepsy** | 11956 | 1672 (12.3) | 3144 | 532 (14.5) | 403 | 69 (14.6) | 195 | 43 (18.1) | 13 | 1 (7.1) |
| **Multiple sclerosis, transv myelitis** | 2500 | 290 (10.4) | 800 | 128 (13.8) | 94 | 17 (15.3) | 41 | 6 (12.8) | 3 | 0 |
| **Muscular dystrophy** | 176 | 31 (15.0) | 56 | 13 (18.8) | 9 | 1 (10.0) | 3 | 1 (25.0) | 0 | 0 |
| **Peripheral neuropathy** | 83764 | 11669 (12.2) | 23842 | 3416 (12.5) | 3469 | 515 (12.9) | 1950 | 340 (14.8) | 172 | 28 (14.0) |
| **Spina bifida, congen neurologic** | 442 | 40 (8.3) | 153 | 15 (8.9) | 30 | 1 (3.2) | 8 | 0 | 3 | 0 |
| **Traumatic brain injury** | 3206 | 270 (7.8) | 948 | 92 (8.8) | 155 | 8 (4.9) | 58 | 7 (10.8) | 4 | 0 |
| **Psychiatric disorder** | 270338 | 23033 (7.9) | 74375 | 6125 (7.6) | 13803 | 1050 (7.1) | 5724 | 547 (8.7) | 549 | 51 (8.5) |
| **Anxiety disorders** | 200375 | 15420 (7.1) | 56117 | 4134 (6.9) | 10683 | 727 (6.4) | 4366 | 380 (8.0) | 418 | 34 (7.5) |
| **ADHD / Conduct disorder** | 13981 | 531 (3.7) | 4192 | 138 (3.2) | 880 | 23 (2.5) | 302 | 17 (5.3) | 40 | 0 |
| **Bipolar disorder** | 52654 | 4561 (8.0) | 14184 | 1337 (8.6) | 2525 | 223 (8.1) | 1053 | 121 (10.3) | 117 | 10 (7.9) |
| **Depression** | 168837 | 14509 (7.9) | 46757 | 3869 (7.6) | 8468 | 665 (7.3) | 3537 | 325 (8.4) | 335 | 24 (6.7) |
| **Depressive disorders** | 152255 | 13147 (7.9) | 42177 | 3522 (7.7) | 7594 | 592 (7.2) | 3153 | 281 (8.2) | 297 | 21 (6.6) |
| **PTSD** | 133003 | 9623 (6.7) | 36751 | 2493 (6.4) | 7007 | 433 (5.8) | 2817 | 216 (7.1) | 269 | 18 (6.3) |
| **Schizophrenia / psychosis** | 24982 | 2516 (9.1) | 6050 | 815 (11.9) | 933 | 100 (9.7) | 398 | 57 (12.5) | 30 | 4 (11.8) |
| **Comorbidities, other ungrouped** |  |  |  |  |  |  |  |  |  |  |
| **Alzheimer's / dementia** | 32723 | 7800 (19.2) | 9584 | 3133 (24.6) | 874 | 324 (27.0) | 640 | 234 | 59 | 17 (22.4) |
| **Anemia** | 84296 | 15178 (15.3) | 23690 | 4830 (16.9) | 3147 | 658 (17.3) | 1798 | 400 | 141 | 35 (19.9) |
| **Asthma** | 32363 | 2801 (8.0) | 10061 | 769 (7.1) | 2334 | 200 (7.9) | 1071 | 96 | 102 | 5 (4.7) |
| **Chronic kidney disease** | 101711 | 17372 (14.6) | 29671 | 5561 (15.8) | 4102 | 765 (15.7) | 2332 | 490 | 177 | 41 (18.8) |
| **COPD / Bronchiectasis** | 79929 | 22071 (21.6) | 20113 | 5606 (21.8) | 3523 | 1196 (25.3) | 1846 | 645 | 154 | 57 (27.0) |
| **Diabetes** | 172941 | 23075 (11.8) | 47728 | 6262 (11.6) | 7300 | 969 (11.7) | 3730 | 567 | 309 | 62 (16.7) |
| **HIV / AIDS** | 5704 | 470 (7.6) | 1342 | 101 (7.0) | 342 | 31 (8.3) | 149 | 28 | 13 | 1 (7.1) |
| **Hypothyroidism** | 47087 | 6493 (12.1) | 13468 | 1970 (12.8) | 2103 | 268 (11.3) | 1096 | 186 | 91 | 18 (16.5) |
| **Tobacco use** | 107301 | 14241 (11.7) | 24860 | 3234 (11.5) | 4975 | 786 (13.6) | 1780 | 339 | 185 | 35 (15.9) |

**sTable 5.** **Death among patients with different test results for viral infection.** Numbers in parentheses indicate the percentages of patients with that variable/characteristic who died within 4 weeks of the index date.

| **Virus test result** | **All negative** | | **SARS-CoV-2 only** | | **Influenza only** | | **RSV only** | | **Multiple tests** | |
| --- | --- | --- | --- | --- | --- | --- | --- | --- | --- | --- |
| **Death within 28 days** | **No** | **Yes** | **No** | **Yes** | **No** | **Yes** | **No** | **Yes** | **No** | **Yes** |
| **Total, N (%)** | 607906 | 12528 (2.0) | 167429 | 3163 (1.9) | 30222 | 232 (0.8) | 13059 | 148 (1.1) | 1281 | 19 (1.5) |
| **Age ranges** |  |  |  |  |  |  |  |  |  |  |
| **17-<40** | 72620 | 97 (0.1) | 21198 | 11 (0.1) | 5813 | 0 | 1409 | 0 | 204 | 0 |
| **40-<45** | 30667 | 56 (0.2) | 9564 | 4 (0.0) | 2109 | 0 | 580 | 0 | 76 | 0 |
| **45-<50** | 27194 | 68 (0.2) | 8622 | 16 (0.2) | 1754 | 2 (0.1) | 596 | 0 | 62 | 0 |
| **50-<55** | 48734 | 209 (0.4) | 15033 | 52 (0.3) | 2892 | 9 (0.3) | 1137 | 2 (0.2) | 115 | 0 |
| **55-<60** | 51907 | 351 (0.7) | 15095 | 66 (0.4) | 2840 | 7 (0.2) | 1142 | 3 (0.3) | 122 | 0 |
| **60-<65** | 72074 | 921 (1.3) | 18290 | 170 (0.9) | 3676 | 9 (0.9) | 1500 | 7 (0.5) | 152 | 2 (1.3) |
| **65-<70** | 72992 | 1377 (1.9) | 18097 | 321 (1.7) | 3106 | 27 (0.9) | 1476 | 15 (1.0) | 140 | 2 (1.4) |
| **70-<75** | 109852 | 2952 (2.6) | 27509 | 715 (2.5) | 3987 | 57 (1.4) | 2349 | 36 (1.5) | 196 | 5 (2.5) |
| **75-<80** | 66133 | 2247 (3.3) | 17929 | 599 (3.2) | 2411 | 39 (1.6) | 1617 | 27 (1.6) | 126 | 4 (3.1) |
| **>=80** | 55659 | 4250 (7.1) | 16071 | 1209 (7.0) | 1631 | 82 (4.8) | 1251 | 58 (4.4) | 88 | 6 (6.4) |
| **Unknown** | 74 | 0 | 21 | 0 | 3 | 0 | 2 | 0 | 0 | 0 |
| **Race** |  |  |  |  |  |  |  |  |  |  |
| **American Indian or Alaska Native** | 4896 | 76 (1.5) | 1524 | 24 (1.6) | 272 | 0 | 113 | 0 | 6 | 0 |
| **Asian** | 6315 | 67 (1.0) | 2280 | 17 (0.7) | 344 | 2 (0.6) | 165 | 0 | 15 | 0 |
| **Black or African American** | 140416 | 2128 (1.5) | 37162 | 478 (1.3) | 7556 | 31 (0.4) | 2620 | 20 (0.8) | 240 | 0 |
| **More than one race** | 6223 | 92 (1.5) | 1744 | 27 (1.5) | 326 | 2 (0.6) | 133 | 0 | 9 | 0 |
| **Native Hawaiian or Pacific Islander** | 5253 | 81 (1.5) | 1609 | 26 (1.6) | 306 | 1 (0.3) | 133 | 1 (0.7) | 9 | 0 |
| **White** | 399126 | 9248 (2.3) | 110005 | 2360 (2.1) | 19019 | 184 (1.0) | 8924 | 119 (1.3) | 900 | 17 (1.9) |
| **Unknown** | 45677 | 836 (1.8) | 13105 | 231 (1.7) | 2399 | 12 (0.5) | 971 | 8 (0.8) | 102 | 2 (1.9) |
| **Sex** |  |  |  |  |  |  |  |  |  |  |
| **F** | 68509 | 354 (0.5) | 20186 | 65 (0.3) | 4081 | 8 (0.2) | 1701 | 5 (0.3) | 160 | 0 |
| **M** | 539323 | 12174 (2.2) | 147223 | 3098 (2.1) | 26138 | 224 (0.8) | 11357 | 143 (1.2) | 1121 | 19 (1.7) |
| **Unknown** | 74 | 0 | 20 | 0 | 3 | 0 | 1 | 0 | 0 | 0 |
| **Ethnicity (Hispanic/Latino)** |  |  |  |  |  |  |  |  |  |  |
| **Hispanic or Latino** | 53380 | 896 (1.7) | 17680 | 317 (1.8) | 3382 | 22 (0.6) | 1285 | 13 (1.0) | 141 | 2 (1.4) |
| **Not Hispanic or Latino** | 522923 | 11026 (2.1) | 140948 | 2714 (1.9) | 25285 | 203 (0.8) | 11150 | 126 (1.1) | 1068 | 17 (1.6) |
| **Unknown** | 31603 | 606 (1.9) | 8801 | 132 (1.5) | 1555 | 7 (0.4) | 624 | 9 (1.4) | 72 | 0 |
| **Region** |  |  |  |  |  |  |  |  |  |  |
| **Continental** | 94008 | 2038 (2.1) | 28360 | 481 (1.7) | 5514 | 35 (0.6) | 2042 | 18 (0.9) | 186 | 3 (1.6) |
| **Midwest** | 121449 | 2597 (2.1) | 33856 | 629 (1.8) | 6130 | 56 (0.9) | 2716 | 29 (1.1) | 285 | 3 (1.0) |
| **North Atlantic** | 140312 | 2732 (1.9) | 35028 | 630 (1.8) | 6355 | 47 (0.7) | 2981 | 35 (1.2) | 266 | 4 (1.5) |
| **Pacific** | 113830 | 2341 (2.0) | 33484 | 621 (1.8) | 5455 | 48 (0.9) | 2736 | 33 (1.2) | 301 | 6 (2.0) |
| **Southeast** | 138306 | 2820 (2.0) | 36700 | 802 (2.1) | 6768 | 46 (0.7) | 2584 | 33 (1.3) | 243 | 3 (1.2) |
| **Unknown** | 1 | 0 | 1 | 0 | 0 | 0 | 0 | 0 | 0 | 0 |
| **Vaccine manufacturer (SARS-CoV-2)** |  |  |  |  |  |  |  |  |  |  |
| **Janssen** | 32834 | 516 (1.5) | 9302 | 137 (1.5) | 1766 | 13 (0.7) | 618 | 2 (0.3) | 76 | 1 (1.3) |
| **Moderna** | 229751 | 5035 (2.1) | 61576 | 1067 (1.7) | 10676 | 104 (1.0) | 5376 | 64 (1.2) | 480 | 9 (1.8) |
| **Pfizer** | 226403 | 4374 (1.9) | 59192 | 966 (1.6) | 10763 | 78 (0.7) | 4834 | 61 (1.2) | 468 | 4 (0.8) |
| **Unknown including unvaccinated** | 118918 | 2603 (2.1) | 37359 | 993 (2.6) | 7017 | 37 (0.5) | 2231 | 21 (0.9) | 257 | 5 (1.9) |
| **SARS-CoV-2 infection before vaccination** | 24783 | 361 (1.4) | 7993 | 62 (0.8) | 1820 | 15 (0.8) | 761 | 5 (0.7) | 57 | 0 |
| **BMI class** |  |  |  |  |  |  |  |  |  |  |
| **Underweight** | 6948 | 942 (11.9) | 1488 | 210 (12.4) | 196 | 15 (7.1) | 109 | 19 (14.8) | 8 | 0 |
| **Normal** | 101115 | 4426 (4.2) | 25269 | 1099 (4.2) | 4038 | 78 (1.9) | 1838 | 43 (2.3) | 172 | 6 (3.4) |
| **Overweight** | 184619 | 3470 (1.8) | 51443 | 865 (1.7) | 9001 | 71 (0.8) | 3979 | 48 (1.2) | 380 | 8 (2.1) |
| **Mild obesity** | 162186 | 1914 (1.2) | 46228 | 536 (1.1) | 8605 | 46 (0.5) | 3651 | 20 (0.5) | 372 | 3 (0.8) |
| **Moderate obesity** | 84012 | 805 (0.9) | 23930 | 237 (1.0) | 4784 | 14 (0.3) | 2043 | 7 (0.3) | 200 | 1 (0.5) |
| **Severe obesity** | 51720 | 502 (1.0) | 14839 | 144 (1.0) | 2964 | 5 (0.2) | 1256 | 8 (0.6) | 125 | 1 (0.8) |
| **Unknown** | 17306 | 469 (2.6) | 4232 | 72 (1.7) | 634 | 3 (0.5) | 183 | 3 (1.6) | 24 | 0 |
| **Immune-suppressive drugs** | 95228 | 2620 (2.7) | 26374 | 857 (3.1) | 6744 | 75 (1.1) | 4460 | 61 (1.3) | 301 | 8 (2.6) |
| **Cytokine-blocking** | 7398 | 86 (1.1) | 2185 | 61 (2.7) | 395 | 4 (1.0) | 232 | 3 (1.3) | 22 | 0 |
| **Glucocorticoids** | 82760 | 2297 (2.7) | 22400 | 699 (3.0) | 6221 | 68 (1.1) | 4148 | 59 (1.4) | 270 | 5 (1.8) |
| **Leukocyte-inhibitory** | 8262 | 196 (2.3) | 2673 | 136 (4.8) | 350 | 3 (0.8) | 242 | 4 (1.6) | 21 | 4 (16.0) |
| **Lymphocyte-depleting** | 4718 | 271 (5.4) | 1924 | 121 (5.9) | 210 | 6 (2.8) | 176 | 5 (2.8) | 12 | 1 (7.7) |
| **Comorbidities** |  |  |  |  |  |  |  |  |  |  |
| **Comorbidity groups and components** |  |  |  |  |  |  |  |  |  |  |
| **Cancer** | 69199 | 3435 (4.7) | 17908 | 846 (4.5) | 2557 | 55 (2.1) | 1538 | 41 (2.6) | 139 | 5 (3.5) |
| **Breast cancer** | 2770 | 66 (2.3) | 677 | 10 (1.5) | 113 | 0 | 73 | 1 (1.4) | 9 | 0 |
| **Colorectal cancer** | 7461 | 409 (5.2) | 1841 | 100 (5.2) | 251 | 10 (3.8) | 151 | 4 (2.6) | 12 | 0 |
| **Endometrial cancer** | 169 | 6 (3.4) | 46 | 0 | 9 | 0 | 6 | 0 | 0 | 0 |
| **Leukemias / Lymphomas** | 9873 | 536 (5.1) | 3095 | 196 (6.0) | 425 | 8 (1.8) | 268 | 6 (2.2) | 21 | 1 (4.5) |
| **Lung cancer** | 11081 | 1190 (9.7) | 2360 | 258 (9.9) | 385 | 13 (3.3) | 229 | 10 (4.2) | 22 | 3 (12.0) |
| **Prostate cancer** | 39575 | 1351 (3.3) | 10390 | 365 (3.4) | 1439 | 26 (1.8) | 842 | 22 (2.5) | 77 | 2 (2.5) |
| **Chemotherapy** | 7146 | 855 (10.7) | 1616 | 187 (10.4) | 192 | 16 (7.7) | 149 | 7 (4.5) | 6 | 1 (14.3) |
| **Cardiovascular disease** | 436334 | 10425 (2.3) | 119759 | 2780 (2.3) | 19844 | 206 (1.0) | 9799 | 137 (1.4) | 887 | 17 (1.9) |
| **Atrial fibrillation** | 60931 | 2830 (4.4) | 16478 | 866 (5.0) | 2265 | 63 (2.7) | 1526 | 51 (3.2) | 119 | 6 (4.8) |
| **Heart failure** | 59479 | 3226 (5.1) | 16450 | 1004 (5.8) | 2335 | 77 (3.2) | 1522 | 66 (4.2) | 113 | 9 (7.4) |
| **Hyperlipidemia** | 310177 | 6971 (2.2) | 87718 | 1990 (2.2) | 14473 | 136 (0.9) | 7261 | 98 (1.3) | 674 | 13 (1.9) |
| **Hypertension** | 350830 | 9039 (2.5) | 95009 | 2474 (2.5) | 15434 | 177 (1.1) | 7854 | 122 (1.5) | 665 | 16 (2.3) |
| **Vascular disease** | 152386 | 5622 (3.6) | 40992 | 1636 (3.8) | 5859 | 134 (2.2) | 3355 | 86 (2.5) | 275 | 9 (3.2) |
| **Acute myocardial infarction** | 9815 | 681 (6.5) | 3518 | 252 (6.7) | 464 | 24 (4.9) | 290 | 16 (5.2) | 14 | 2 (12.5) |
| **Ischemic heart disease** | 115016 | 4207 (3.5) | 31478 | 1244 (3.8) | 4523 | 111 (2.4) | 2671 | 69 (2.5) | 223 | 7 (3.0) |
| **Peripheral vascular disease** | 43226 | 1950 (4.3) | 11167 | 603 (5.1) | 1521 | 43 (2.7) | 879 | 29 (3.2) | 76 | 5 (6.2) |
| **Stroke / TIA** | 28676 | 1156 (3.9) | 8096 | 388 (4.6) | 1027 | 20 (1.9) | 630 | 23 (3.5) | 49 | 3 (5.8) |
| **Impaired function / mobility** | 31376 | 1812 (5.5) | 8477 | 618 (6.8) | 949 | 27 (2.8) | 566 | 39 (6.4) | 50 | 4 (7.4) |
| **Mobility impairments** | 12965 | 509 (3.8) | 3506 | 151 (4.1) | 364 | 8 (2.2) | 223 | 11 (4.7) | 23 | 3 (11.5) |
| **Pressure / chronic ulcers** | 20611 | 1422 (6.5) | 5468 | 507 (8.5) | 606 | 23 (3.7) | 369 | 34 (8.4) | 27 | 2 (6.9) |
| **Spinal cord injury** | 2462 | 112 (4.4) | 669 | 28 (4.0) | 81 | 1 (1.2) | 48 | 2 (4.0) | 5 | 0 |
| **Liver disease** | 54340 | 1714 (3.1) | 14647 | 490 (3.2) | 2479 | 38 (1.5) | 1123 | 24 (2.1) | 115 | 3 (2.5) |
| **Liver cirrhosis and other non-viral** | 44140 | 1474 (3.2) | 12156 | 431 (3.4) | 2031 | 31 (1.5) | 942 | 22 (2.3) | 91 | 3 (3.2) |
| **Viral hepatitis** | 15574 | 478 (3.0) | 3927 | 117 (2.9) | 633 | 14 (2.2) | 267 | 7 (2.6) | 34 | 0 |
| **Neurologic disease** | 108113 | 2769 (2.5) | 30640 | 868 (2.8) | 4548 | 60 (1.3) | 2513 | 37 (1.5) | 211 | 5 (2.3) |
| **Cerebral palsy** | 49 | 2 (3.9) | 11 | 1 (8.3) | 0 | 0 | 2 | 0 | 0 | 0 |
| **Epilepsy** | 13190 | 438 (3.2) | 3546 | 130 (3.5) | 463 | 9 (1.9) | 232 | 6 (2.5) | 13 | 1 (7.1) |
| **Multiple sclerosis / Transverse myelitis** | 2738 | 52 (1.9) | 914 | 14 (1.5) | 108 | 3 (2.7) | 45 | 2 (4.3) | 3 | 0 |
| **Muscular dystrophy** | 201 | 6 (2.9) | 62 | 7 (10.1) | 10 | 0 | 4 | 0 | 0 | 0 |
| **Peripheral neuropathy** | 93076 | 2357 (2.5) | 26509 | 749 (2.7) | 3935 | 49 (1.2) | 2258 | 32 (1.4) | 196 | 4 (2.0) |
| **Spina bifida / congenital neurologic** | 472 | 10 (2.1) | 163 | 5 (3.0) | 31 | 0 | 8 | 0 | 2 | 1 (33.3) |
| **Traumatic brain injury** | 3407 | 69 (2.0) | 1020 | 20 (1.9) | 162 | 1 (0.6) | 65 | 0 | 4 | 0 |
| **Psychiatric disorder** | 289037 | 4334 (1.5) | 79272 | 1228 (1.5) | 14754 | 99 (0.7) | 6206 | 65 (1.0) | 591 | 9 (1.5) |
| **Anxiety disorders** | 213038 | 2757 (1.3) | 59491 | 760 (1.3) | 11342 | 68 (0.6) | 4701 | 45 (0.9) | 448 | 4 (0.9) |
| **ADHD / Conduct disorder** | 14442 | 70 (0.5) | 4313 | 17 (0.4) | 901 | 2 (0.2) | 318 | 1 (0.3) | 40 | 0 |
| **Bipolar disorder** | 56460 | 755 (1.3) | 15304 | 217 (1.4) | 2728 | 20 (0.7) | 1161 | 13 (1.1) | 127 | 0 |
| **Depression** | 180846 | 2500 (1.4) | 49907 | 719 (1.4) | 9069 | 64 (0.7) | 3830 | 32 (0.8) | 351 | 8 (2.2) |
| **Depressive disorders** | 163163 | 2239 (1.4) | 45050 | 649 (1.4) | 8128 | 58 (0.7) | 3409 | 25 (0.7) | 312 | 6 (1.9) |
| **PTSD** | 141128 | 1498 (1.1) | 38834 | 410 (1.0) | 7408 | 32 (0.4) | 3013 | 20 (0.7) | 285 | 2 (0.7) |
| **Schizophrenia / psychosis** | 27057 | 441 (1.6) | 6730 | 135 (2.0) | 1022 | 11 (1.1) | 446 | 9 (2.0) | 34 | 0 |
| **Comorbidities, other ungrouped** |  |  |  |  |  |  |  |  |  |  |
| **Alzheimer's / dementia** | 37578 | 2945 (7.3) | 11711 | 1006 (7.9) | 1131 | 67 (5.6) | 822 | 52 (5.9) | 70 | 6 (7.9) |
| **Anemia** | 94898 | 4576 (4.6) | 27084 | 1436 (5.0) | 3705 | 100 (2.6) | 2122 | 76 (3.5) | 166 | 10 (5.7) |
| **Asthma** | 34773 | 391 (1.1) | 10714 | 116 (1.1) | 2520 | 14 (0.6) | 1161 | 6 (0.5) | 107 | 0 |
| **Chronic kidney disease** | 114423 | 4660 (3.9) | 33718 | 1514 (4.3) | 4764 | 103 (2.1) | 2734 | 88 (3.1) | 209 | 9 (4.1) |
| **COPD / Bronchiectasis** | 98119 | 3881 (3.8) | 24669 | 1050 (4.1) | 4621 | 98 (2.1) | 2425 | 66 (2.6) | 204 | 7 (3.3) |
| **Diabetes** | 191048 | 4968 (2.5) | 52565 | 1425 (2.6) | 8169 | 100 (1.2) | 4224 | 73 (1.7) | 360 | 11 (3.0) |
| **HIV / AIDS** | 6070 | 104 (1.7) | 1419 | 24 (1.7) | 370 | 3 (0.8) | 175 | 2 (1.1) | 14 | 0 |
| **Hypothyroidism** | 51940 | 1640 (3.1) | 14931 | 507 (3.3) | 2337 | 34 (1.4) | 1255 | 27 (2.1) | 105 | 4 (3.7) |
| **Tobacco use** | 118797 | 2745 (2.3) | 27442 | 652 (2.3) | 5703 | 58 (1.0) | 2092 | 27 (1.3) | 216 | 4 (1.8) |

**sTable 6**. **Associations of selected clinical features with outcomes of hypoxemia or death, with and without adjustment for age**. All patients with viral infection were included and analyzed together. Multiple logistic regression was used with the type of viral infection as a 4-part independent variable (influenza, RSV, or multiple, relative to SARS-CoV-2 as the referent) and the listed characteristic as another independent variable (yes/no), with and without age as a third independent variable modeled as a 3-part spline. For results stratified by infection, see sTables 7 and 8. aOR = adjusted odds ratio; CI = 95% confidence interval.

|  | **Outcome = Hypoxemia within 14 days** | |  | **Outcome = Death within 28 days** | |
| --- | --- | --- | --- | --- | --- |
| **Covariate/Comorbidity** | **aOR (95% CI)** | **Adjusted for age**  **aOR (95% CI)** |  | **aOR (95% CI)** | **Adjusted for age**  **aOR (95% CI)** |
| **Sex (ref = male)** | 0.33 (0.31 - 0.36) | 0.76 (0.70 - 0.82) |  | 0.16 (0.13 - 0.20) | 0.49 (0.39 - 0.62) |
| **Underweight (ref = normal BMI)** | 2.75 (2.48 - 3.04) | 2.42 (2.17 - 2.68) |  | 3.43 (2.96 - 3.97) | 3.04 (2.61 - 3.54) |
| **Mobility/functional impairment** | 3.92 (3.74 - 4.11) | 2.63 (2.50 - 2.76) |  | 4.65 (4.27 - 5.07) | 2.83 (2.60 - 3.09) |
| **Immune-suppressive drugs** | 2.83 (2.74 - 2.93) | 2.60 (2.51 - 2.69) |  | 1.93 (1.79 - 2.07) | 1.78 (1.65 - 1.92) |
| **Tobacco use** | 1.78 (1.71 - 1.84) | 2.22 (2.14 - 2.31) |  | 1.33 (1.22 - 1.44) | 1.99 (1.82 - 2.16) |
| **Alzheimers / dementia** | 4.58 (4.39 - 4.77) | 2.40 (2.30 - 2.51) |  | 6.47 (6.01 - 6.96) | 2.69 (2.48 - 2.90) |
| **Anemia** | 3.05 (2.95 - 3.16) | 2.03 (1.96 - 2.11) |  | 4.43 (4.14 - 4.74) | 2.61 (2.43 - 2.79) |
| **Cancer** | 2.15 (2.07 - 2.24) | 1.24 (1.19 - 1.30) |  | 3.06 (2.84 - 3.30) | 1.66 (1.54 - 1.79) |
| **Cardiovascular disease** | 2.92 (2.79 - 3.06) | 1.20 (1.14 - 1.26) |  | 3.00 (2.71 - 3.33) | 1.06 (0.96 - 1.18) |
| **Chronic kidney disease** | 2.92 (2.82 - 3.01) | 1.89 (1.83 - 1.96) |  | 3.75 (3.51 - 4.01) | 2.23 (2.08 - 2.38) |
| **COPD** | 5.12 (4.96 - 5.29) | 3.35 (3.23 - 3.46) |  | 2.97 (2.77 - 3.19) | 1.82 (1.69 - 1.95) |
| **Diabetes** | 1.95 (1.89 - 2.01) | 1.29 (1.25 - 1.33) |  | 1.82 (1.71 - 1.95) | 1.23 (1.15 - 1.32) |
| **Liver disease** | 1.47 (1.40 - 1.54) | 1.52 (1.45 - 1.60) |  | 1.94 (1.77 - 2.12) | 2.38 (2.16 - 2.61) |
| **Neurologic disease** | 1.92 (1.85 - 1.98) | 1.47 (1.41 - 1.52) |  | 1.69 (1.57 - 1.82) | 1.30 (1.20 - 1.40) |
| **Psychiatric disease** | 0.89 (0.86 - 0.92) | 1.32 (1.28 - 1.36) |  | 0.72 (0.67 - 0.77) | 1.21 (1.12 - 1.29) |
| **Vascular disease** | 3.00 (2.91 - 3.10) | 1.62 (1.57 - 1.67) |  | 3.45 (3.23 - 3.69) | 1.64 (1.53 - 1.76) |

**sTable 7**. **Associations of selected clinical features with the outcome of hypoxemia within 14 days of the index date, stratified by type of viral infection**. Logistic regression was used with the listed characteristic as the only independent variable. Data are shown as odds ratios and 95% confidence intervals.

| **Covariate/Comorbidity** | **All tests negative** | **All patients with a positive test** | **SARS-CoV-2 only** | **Influenza only** | **RSV only** | **Multiple positive** |
| --- | --- | --- | --- | --- | --- | --- |
| **Sex (ref = male)** | 0.43 (0.42 - 0.45) | 0.33 (0.31 - 0.36) | 0.30 (0.27 - 0.32) | 0.47 (0.40 - 0.55) | 0.47 (0.37 - 0.58) | 0.45 (0.20 - 0.98) |
| **Underweight (ref = normal BMI)** | 2.29 (2.17 - 2.42) | 2.75 (2.48 - 3.04) | 2.70 (2.42 - 3.03) | 2.53 (1.85 - 3.47) | 3.52 (2.41 - 5.15) | 10.7 (2.40 - 47.7) |
| **Mobility/functional impairment** | 2.64 (2.56 - 2.71) | 3.91 (3.73 - 4.10) | 3.93 (3.73 - 4.14) | 4.23 (3.63 - 4.93) | 3.34 (2.74 - 4.07) | 4.06 (2.13 - 7.71) |
| **Immune-suppressive drugs** | 2.36 (2.31 - 2.41) | 2.80 (2.71 - 2.90) | 2.58 (2.48 - 2.68) | 4.14 (3.80 - 4.52) | 3.28 (2.91 - 3.70) | 2.84 (1.91 - 4.23) |
| **Tobacco use** | 1.49 (1.46 - 1.52) | 1.77 (1.71 - 1.84) | 1.65 (1.58 - 1.72) | 2.34 (2.13 - 2.56) | 2.20 (1.92 - 2.52) | 2.43 (1.58 - 3.73) |
| **Alzheimers / dementia** | 2.68 (2.61 - 2.75) | 4.56 (4.37 - 4.75) | 4.57 (4.37 - 4.78) | 4.98 (4.35 - 5.70) | 4.20 (3.57 - 4.94) | 3.39 (1.90 - 6.04) |
| **Anemia** | 2.17 (2.12 - 2.21) | 3.05 (2.95 - 3.16) | 3.07 (2.96 - 3.19) | 3.08 (2.80 - 3.40) | 2.76 (2.42 - 3.14) | 3.33 (2.15 - 5.15) |
| **Cancer** | 1.79 (1.75 - 1.83) | 2.16 (2.07 - 2.25) | 2.15 (2.05 - 2.25) | 2.24 (1.99 - 2.53) | 2.04 (1.75 - 2.37) | 2.55 (1.57 - 4.13) |
| **Cardiovascular disease** | 2.14 (2.09 - 2.19) | 2.92 (2.79 - 3.06) | 2.82 (2.68 - 2.97) | 3.33 (2.96 - 3.75) | 3.18 (2.63 - 3.85) | 4.45 (2.36 - 8.39) |
| **Chronic kidney disease** | 2.10 (2.06 - 2.14) | 2.92 (2.82 - 3.01) | 2.94 (2.84 - 3.05) | 2.83 (2.58 - 3.10) | 2.77 (2.45 - 3.13) | 3.25 (2.14 - 4.92) |
| **COPD** | 4.05 (3.98 - 4.13) | 5.13 (4.96 - 5.30) | 4.73 (4.56 - 4.91) | 7.23 (6.62 - 7.90) | 6.14 (5.43 - 6.94) | 6.83 (4.55 - 10.2) |
| **Diabetes** | 1.63 (1.60 - 1.66) | 1.95 (1.89 - 2.01) | 1.93 (1.87 - 2.00) | 2.00 (1.83 - 2.18) | 1.92 (1.70 - 2.16) | 3.45 (2.33 - 5.12) |
| **Liver disease** | 1.23 (1.20 - 1.27) | 1.47 (1.40 - 1.54) | 1.45 (1.37 - 1.53) | 1.51 (1.33 - 1.73) | 1.58 (1.32 - 1.90) | 1.47 (0.81 - 2.67) |
| **Neurologic disease** | 1.52 (1.49 - 1.56) | 1.92 (1.85 - 1.99) | 1.90 (1.82 - 1.97) | 2.00 (1.82 - 2.21) | 1.96 (1.72 - 2.24) | 1.85 (1.18 - 2.90) |
| **Psychiatric disease** | 0.78 (0.77 - 0.80) | 0.89 (0.86 - 0.92) | 0.90 (0.87 - 0.93) | 0.84 (0.77 - 0.91) | 0.89 (0.79 - 1.00) | 0.96 (0.65 - 1.41) |
| **Vascular disease** | 2.25 (2.21 - 2.29) | 3.00 (2.91 - 3.10) | 2.94 (2.84 - 3.05) | 3.54 (3.24 - 3.86) | 2.78 (2.46 - 3.13) | 2.98 (2.00 - 4.44) |
| **Age categories** |  |  |  |  |  |  |
| **<50** | 0.18 (0.17 - 0.19) | 0.12 (0.11 - 0.13) | 0.12 (0.11 - 0.14) | 0.08 (0.07 - 0.10) | 0.12 (0.09 - 0.17) | 0.06 (0.02 - 0.27) |
| **50-<60** | 0.47 (0.45 - 0.48) | 0.36 (0.34 - 0.39) | 0.37 (0.35 - 0.40) | 0.33 (0.28 - 0.38) | 0.35 (0.27 - 0.45) | 0.38 (0.17 - 0.86) |
| **60-<70 (referent)** | Referent | Referent | Referent | Referent | Referent | Referent |
| **70-<80** | 1.47 (1.44 - 1.51) | 1.69 (1.63 - 1.76) | 1.74 (1.66 - 1.82) | 1.69 (1.52 - 1.87) | 1.40 (1.21 - 1.62) | 2.21 (1.34 - 3.64) |
| **>=80** | 2.12 (2.06 - 2.18) | 2.65 (2.52 - 2.78) | 2.80 (2.65 - 2.95) | 2.34 (2.04 - 2.70) | 2.02 (1.69 - 2.43) | 3.31 (1.77 - 6.21) |

**sTable 8**. **Associations of selected clinical features with the outcome of death within 4 weeks of the index date, stratified by type of viral infection**. Logistic regression was used with the listed characteristic as the only independent variable. Data are shown as odds ratios and 95% confidence intervals. ND = not done, due to insufficient numbers of events.

| **Covariate/Comorbidity** | **All tests negative** | **All patients with a positive test** | **SARS-CoV-2 only** | **Influenza only** | **RSV only** | **Multiple positive** |
| --- | --- | --- | --- | --- | --- | --- |
| **Sex (ref = male)** | 0.23 (0.21 - 0.25) | 0.16 (0.13 - 0.20) | 0.15 (0.12 - 0.20) | 0.23 (0.11 - 0.46) | 0.23 (0.10 - 0.57) | ND |
| **Underweight (ref = normal BMI)** | 3.10 (2.88 - 3.34) | 3.46 (2.99 - 4.00) | 3.24 (2.77 - 3.80) | 3.96 (2.24 - 7.01) | 7.45 (4.20 - 13.2) | ND |
| **Mobility/functional impairment** | 3.11 (2.95 - 3.27) | 4.82 (4.42 - 5.25) | 4.55 (4.16 - 4.99) | 4.06 (2.71 - 6.10) | 7.90 (5.43 - 11.5) | 6.57 (2.10 - 20.5) |
| **Immune-suppressive drugs** | 1.42 (1.36 - 1.49) | 1.80 (1.67 - 1.93) | 1.99 (1.84 - 2.15) | 1.66 (1.26 - 2.19) | 1.35 (0.97 - 1.88) | 2.37 (0.94 - 5.94) |
| **Tobacco use** | 1.16 (1.11 - 1.21) | 1.31 (1.21 - 1.42) | 1.33 (1.21 - 1.45) | 1.43 (1.06 - 1.93) | 1.17 (0.77 - 1.78) | 1.32 (0.43 - 4.00) |
| **Alzheimers / dementia** | 4.67 (4.47 - 4.87) | 6.72 (6.25 - 7.22) | 6.20 (5.74 - 6.70) | 10.4 (7.82 - 14.0) | 8.06 (5.71 - 11.4) | 7.98 (2.95 - 21.6) |
| **Anemia** | 3.11 (3.00 - 3.23) | 4.52 (4.23 - 4.84) | 4.31 (4.01 - 4.63) | 5.42 (4.17 - 7.05) | 5.44 (3.93 - 7.53) | 7.46 (2.99 - 18.6) |
| **Cancer** | 2.94 (2.83 - 3.06) | 3.11 (2.88 - 3.35) | 3.05 (2.81 - 3.30) | 3.36 (2.48 - 4.56) | 2.87 (1.99 - 4.13) | 2.93 (1.04 - 8.27) |
| **Cardiovascular disease** | 1.95 (1.86 - 2.04) | 3.05 (2.76 - 3.38) | 2.89 (2.59 - 3.21) | 4.14 (2.75 - 6.23) | 4.14 (2.24 - 7.67) | 3.78 (0.87 - 16.4) |
| **Chronic kidney disease** | 2.55 (2.46 - 2.65) | 3.82 (3.57 - 4.08) | 3.64 (3.39 - 3.91) | 4.27 (3.29 - 5.54) | 5.54 (3.98 - 7.71) | 4.62 (1.85 - 11.5) |
| **COPD** | 2.33 (2.24 - 2.42) | 2.94 (2.74 - 3.16) | 2.88 (2.67 - 3.10) | 4.05 (3.12 - 5.27) | 3.53 (2.54 - 4.90) | 3.08 (1.20 - 7.92) |
| **Diabetes** | 1.43 (1.38 - 1.49) | 1.85 (1.73 - 1.98) | 1.79 (1.67 - 1.92) | 2.05 (1.58 - 2.66) | 2.04 (1.47 - 2.82) | 3.52 (1.40 - 8.82) |
| **Liver disease** | 1.61 (1.53 - 1.70) | 1.95 (1.78 - 2.13) | 1.91 (1.73 - 2.11) | 2.19 (1.54 - 3.11) | 2.06 (1.32 - 3.20) | 1.90 (0.55 - 6.22) |
| **Neurologic disease** | 1.31 (1.26 - 1.37) | 1.72 (1.59 - 1.85) | 1.69 (1.56 - 1.83) | 1.97 (1.47 - 2.65) | 1.40 (0.96 - 2.03) | 1.81 (0.65 - 5.08) |
| **Psychiatric disease** | 0.58 (0.56 - 0.61) | 0.71 (0.67 - 0.77) | 0.71 (0.66 - 0.76) | 0.78 (0.60 - 1.01) | 0.86 (0.62 - 1.20) | 1.05 (0.42 - 2.60) |
| **Vascular disease** | 2.43 (2.35 - 2.52) | 3.52 (3.29 - 3.76) | 3.30 (3.08 - 3.55) | 5.69 (4.37 - 7.39) | 4.01 (2.89 - 5.57) | 3.29 (1.32 - 8.18) |
| **Age categories** |  |  |  |  |  |  |
| **<50** | 0.11 (0.09 - 0.12) | 0.05 (0.04 - 0.08) | 0.06 (0.04 - 0.08) | 0.04 (0.01 - 0.16) | ND | ND |
| **50-<60** | 0.35 (0.32 - 0.39) | 0.30 (0.25 - 0.37) | 0.29 (0.24 - 0.36) | 0.53 (0.29 - 0.95) | 0.30 (0.11 - 0.78) | ND |
| **60-<70 (referent)** | Referent | Referent | Referent | Referent | Referent | Referent |
| **70-<80** | 1.86 (1.77 - 1.96) | 2.22 (2.01 - 2.45) | 2.14 (1.93 - 2.38) | 2.83 (1.92 - 4.15) | 2.15 (1.32 - 3.50) | 2.04 (0.62 - 6.70) |
| **>=80** | 4.82 (4.58 - 5.08) | 5.98 (5.40 - 6.61) | 5.58 (5.01 - 6.20) | 9.47 (6.38 - 14.1) | 6.27 (3.82 - 10.3) | 4.98 (1.37 - 18.0) |
